# Supplementary material for: Infertility-Causing Haploinsufficiency Reveals TRIM28/KAP1 Requirement in Spermatogonia
Source: Stem Cell Reports. 2020 Apr 16;14(5):818–27. doi: 10.1016/j.stemcr.2020.03.013 (PMC7220855; doi:10.1016/j.stemcr.2020.03.013)
Supplement: Document S1. Supplemental Experimental Procedures, Figures S1–S5, and Tables S1 and S2 [file mmc1.pdf]

Stem Cell Reports, Volume *14*

Supplemental Information

Infertility-Causing Haploinsufficiency Reveals TRIM28/KAP1 Requirement in Spermatogonia

Joel H.L. Tan, Heike Wollmann, Ans M.M. van Pelt, Philipp Kaldis, and Daniel M. Messerschmidt

Tan et al. Fig. S1

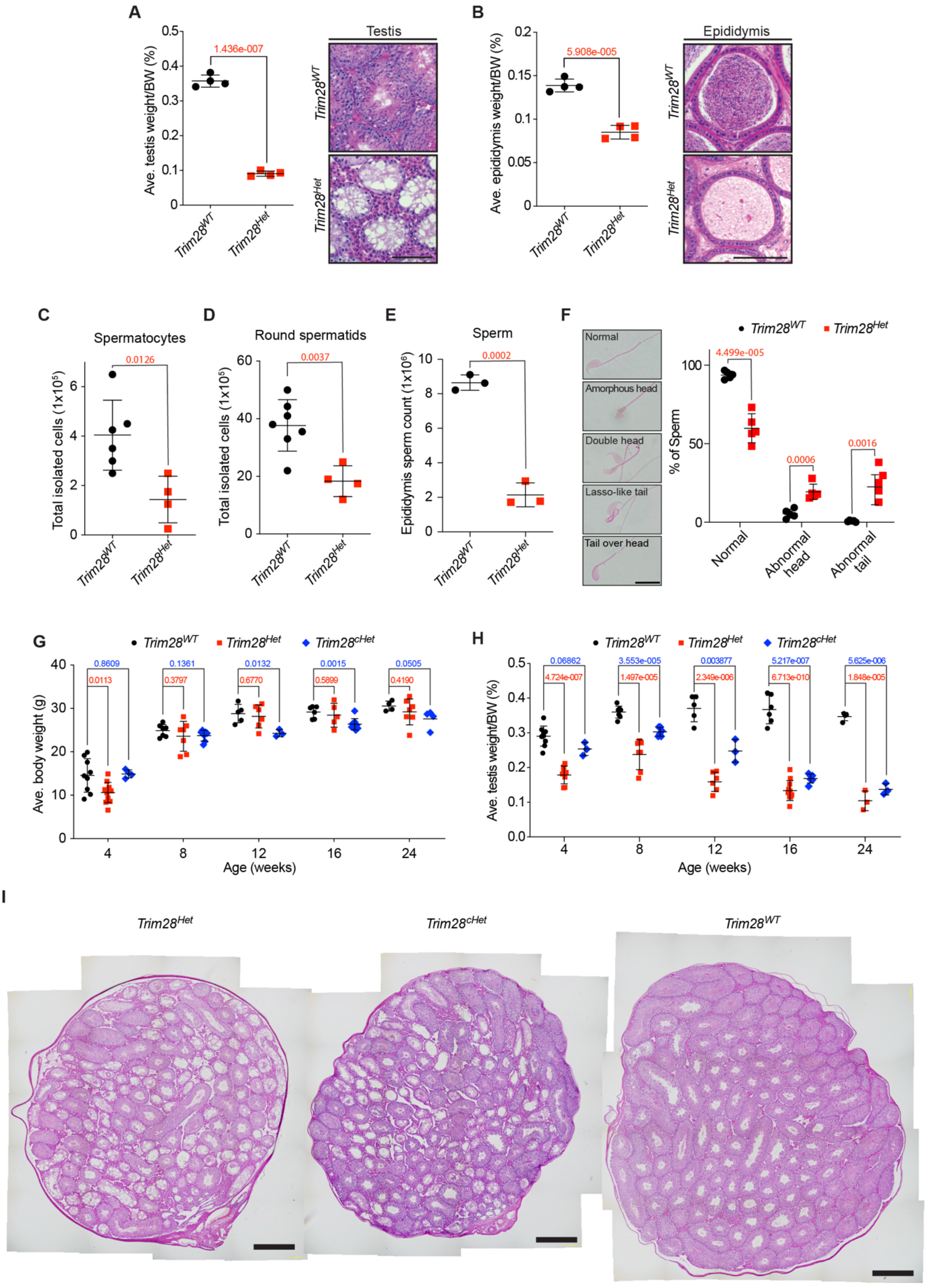

1
2

Figure S1. *Trim28* haploinsufficiency phenotype (Related to Figure 1). (A and B) H&E staining of testis (A) and epididymis (B) sections, and their respective average organ-to-body weight ratio, of males at the end of the mating experiment. Scale bar, 50 µm. SD and p values are indicated; n = 4. (C, D, and E) Cell counts of STAPUT-isolated spermatocytes (C) and round spermatids (D), and epididymal sperm (E) from $Trim28^{WT}$ and $Trim28^{Het}$ males at 16 weeks. SD and p values are indicated; n ≥ 3. (F) Sperm morphological defects and their percentages observed in $Trim28^{Het}$ males at 16 weeks. Scale bar, 5 µm. SD and p values are indicated; n = 5. (G and H) Average body weight (G) and average testis-to-body weight ratio (H) of 4-, 8-, 12-, 16-, and 24-week-old males. SD and p values are indicated; n ≥ 3. (I) H&E staining of whole testis sections from 25-week-old males. Scale bar, 200 µm. Student's t test was used for all significance tests.

Tan et al. Fig. S2

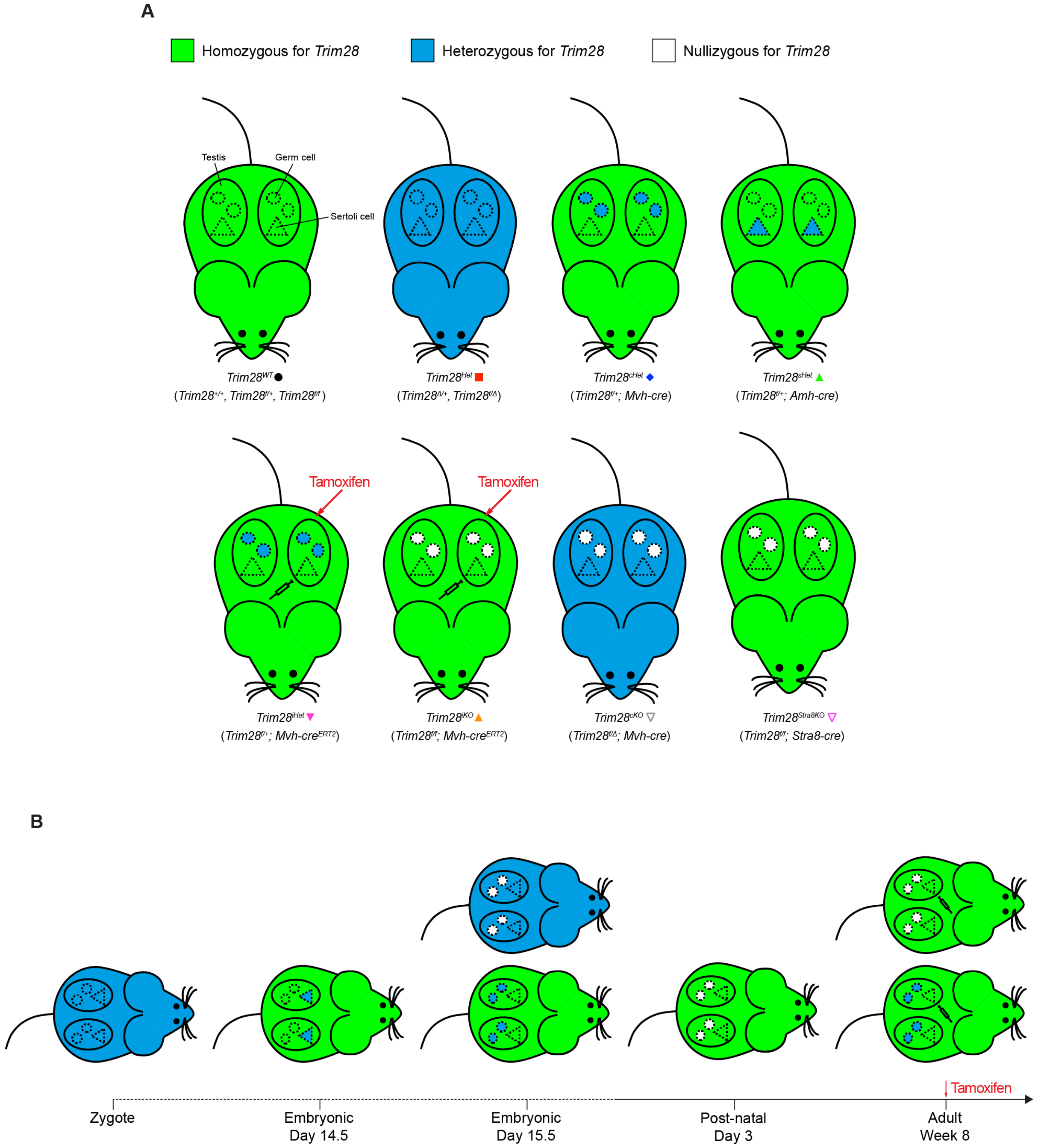

Figure S2. Mouse models. (A) Illustration of mouse models used in the study showing zygosity of *Trim28* in different cell types. Genotypes of mice as indicated. (B) Time points at which deletion of *Trim28* allele(s) occurs for the different mouse models.

Tan et al. Fig. S3

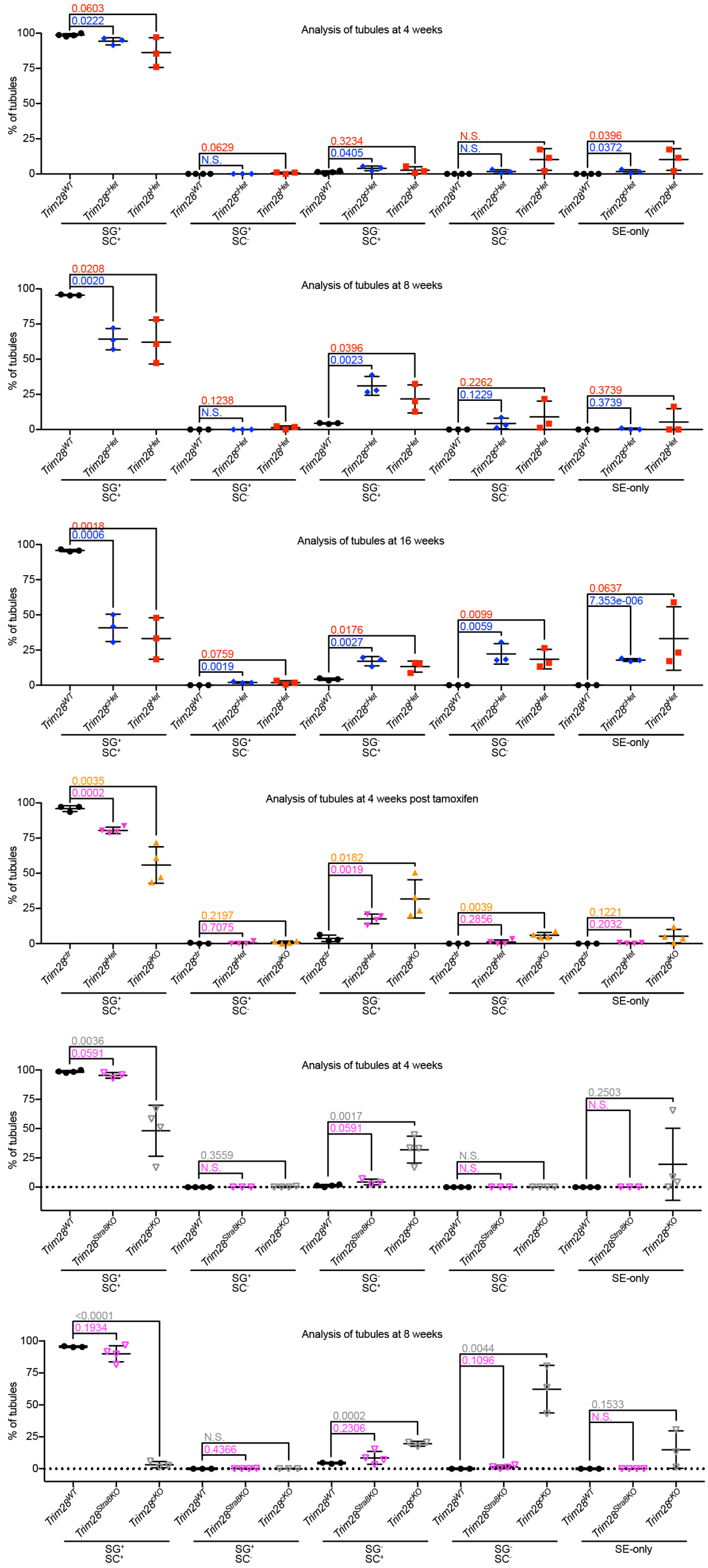

Figure S3. Statistics for sequential loss of germ cells (Related to Figures 2, 5 and 6). Germ cell composition of seminiferous tubules commonly observed in mutant and control testes. For instance, SG^-/SC^+ are tubules that lack SALL4-positive spermatogonia (SG) but contain spermatocytes (SC). All tubules except Sertoli cell-only (SE-only) ones contain spermatids. SD and p values are indicated; $n \geq 3$. Student's t test was used for all significance tests.

Tan et al. Fig. S4

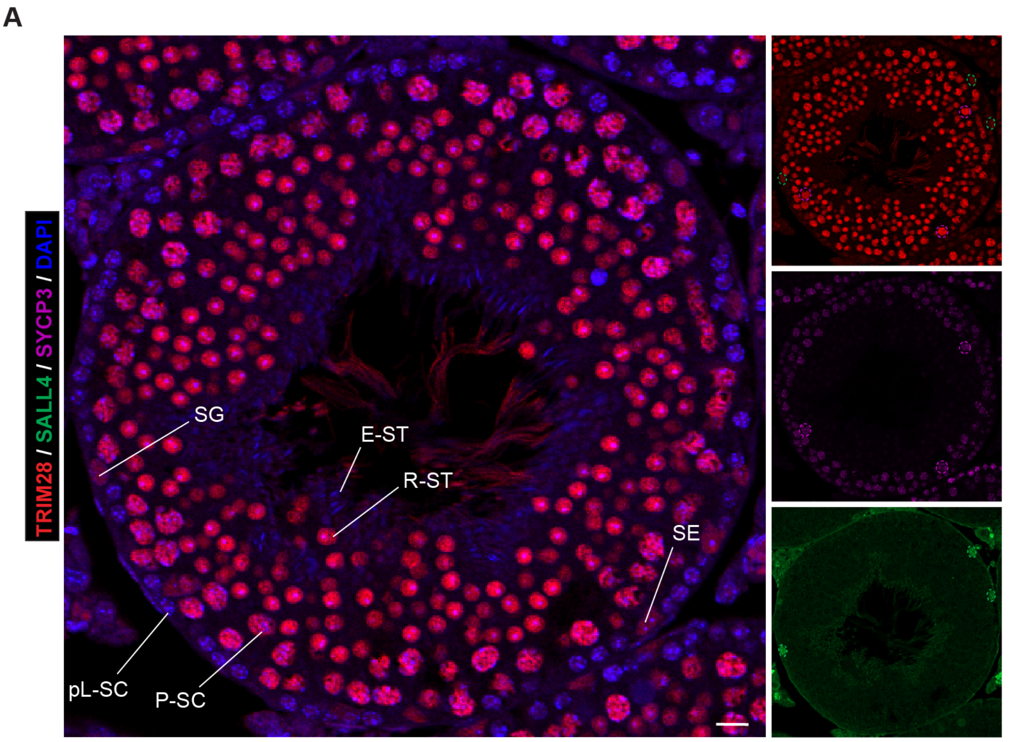

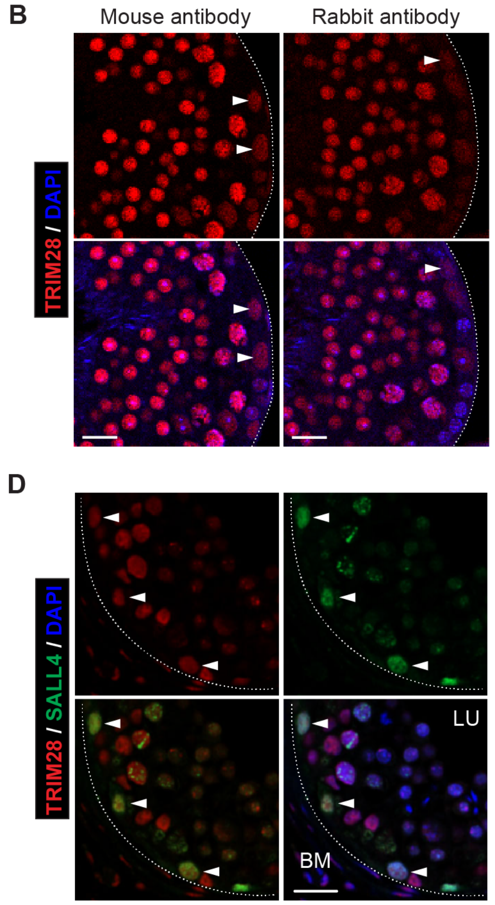

C

	Progenitor	SSC
Trim28	295.5	244.8
Ddx4	201.9	247.3
Id4	6.0	49.4
Gfrα1	5.2	85.4
Etv5	6.2	86.1
Bcl6b	6.9	64.6
Lhx1	0.5	19.4
T	0.3	13.8
Sphk1	8.1	58.7
Nanos2	1.0	4.2
Plzf	42.3	79.1
Utf1	7.9	5.7
Sall4	57.1	95.7
Stra8	133.9	60.8
c-kit	108.2	24.2
Sox9	0.4	1.3
Gapdh	419.0	422.0
Ppia	988.6	1028.0
β-actin	636.8	728.8

FPKM 500 1000

Figure S4. Expression pattern of TRIM28 (Related to Figure 3). (A) Immunofluorescence staining of a Stage 7 adult seminiferous tubule showing TRIM28 expression in germ cells up to round spermatids (R-ST). Scale bar, 20 µm. Spermatocytes (SYCP3-positive) and spermatogonia (SALL4-positive) both express TRIM28 although at varying levels (small panels). (B) Verification of TRIM28 expression in SALL4-positive spermatogonia (arrowhead) using different TRIM28 antibodies: mouse (ab22553, Abcam, left panels) and rabbit (ab10484, Abcam, right panels). Scale bars, 20 µm. (C) RNA-seq data adapted from (Helsel et al. 2017) showing expression level of *Trim28* with respect to other cellular markers in progenitor spermatogonia (ID4-eGFP dim) and SSCs (ID4-eGFP bright). (D) Immunofluorescence staining of adult human seminiferous tubule showing TRIM28 expression in SALL4-positive spermatogonia (arrowhead). Scale bar, 20 µm. BM, basement membrane; LU, lumen.

Tan et al. Fig. S5

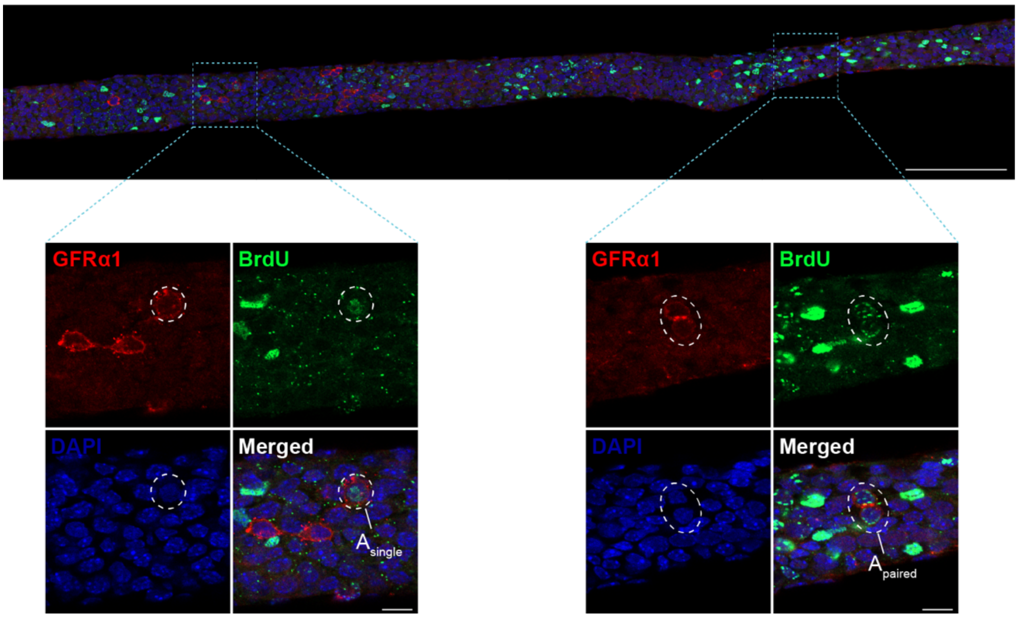

Figure S5. Whole-mount immunofluorescence staining (Related to Figure 4). Representative image of a segment of *Trim28^{WT}* seminiferous tubule 5 days after BrdU injection. Scale bar, 100 µm. Magnified panels show BrdU-positive A_{single} and A_{paired} GFRα1-positive spermatogonia. Scale bar, 10 µm.

Tan et al. Table S1

Gene/Retrotransposon	Sequence	UPL Probe
Trim28	cgcatgtatcaggcatgaag	83
	cttccaggaaagaccttgaaga	
β-actin	aaggccaaccgtgaaaagat	56
	gtggtacgaccagaggcatac	

Table S1. Primers for RT-qPCR.

Tan et al. Table S2

**Primary antibodies**			
**Antibody**	**Company**	**Cat. No.**	**Dilution**
anti-TRIM28	Abcam	ab22553	1:200
anti-TRIM28	Abcam	ab10484	1:200
anti-SALL4	Santa Cruz	sc101147	1:100
anti-SYCP3	Santa Cruz	sc20845	1:100
anti-SOX9	Merck Millipore	ab5535	1:100
anti-BrdU	BD Biosciences	bd555627	1:100
anti-GFRα1	R&D Systems	AF560	1:100
anti-ID4	CalBioreagents	M106	1:100
**Secondary antibodies**			
**Antibody**	**Company**	**Cat. No.**	**Dilution**
Donkey anti-Mouse Alexa Fluor 488	Thermo Fisher	A32766	1:500
Donkey anti-Rabbit Alexa Fluor 594	Thermo Fisher	A32754	1:500
Donkey anti-Goat Alexa Fluor 647	Thermo Fisher	A32849	1:500

Table S2. Antibodies for immunofluorescence staining.

Supplemental Experimental Procedures

Epididymal spermatozoon count

Incisions were made in both caudal epididymides (left and right) before placing them in DSP buffer (0.9% NaCl and 0.05% Triton™ X-100) for 15 min to allow spermatozoa to swim out. A 1:10 dilution of spermatozoa suspension was made before loading both chambers of the hemocytometer (Neubauer Chamber). Spermatozoa were quantified using five squares of each chamber. The counts from both chambers were averaged and the final spermatozoon count was determined using the formula: Spermatozoon count = Dilution × (Count in 5 squares) $\times 0.05 \times 10^6$.

H&E spermatozoa smear

Caudal epididymides were snipped and placed in 1 mL PBS at 37°C for 30 min. Spermatozoa released were centrifuged at 600 × *g* for 10 min and resuspended in a smaller volume of PBS before smearing onto glass slides. Slides were air-dried overnight at room temperature. Prior to H&E staining (haematoxylin step onwards), slides were rehydrated in water. Spermatozoon morphology was analysed by counting at least ten 40X magnification fields of spermatozoa. A minimum of three mice were examined for each genotype.
