# Supplementary material for: Infertility-Causing Haploinsufficiency Reveals TRIM28/KAP1 Requirement in Spermatogonia
Source: Stem Cell Reports. 2020 Apr 16;14(5):818–27. doi: 10.1016/j.stemcr.2020.03.013 (PMC7220855; doi:10.1016/j.stemcr.2020.03.013)
Supplement: Document S2. Article plus Supplemental Information [file mmc2.pdf]

Stem Cell Reports
Article
OPEN ACCESS

Infertility-Causing Haploinsufficiency Reveals TRIM28/KAP1 Requirement in Spermatogonia

Joel H.L. Tan,[1,2] Heike Wollmann,[1] Ans M.M. van Pelt,[3] Philipp Kaldis,[1] and Daniel M. Messerschmidt[1,*]
[1]Institute of Molecular and Cell Biology (IMCB), Agency for Science, Technology and Research (A*STAR), Singapore 138673, Singapore
[2]NUS Graduate School for Integrative Sciences and Engineering (NGS), National University of Singapore, Singapore 119077, Singapore
[3]Center for Reproductive Medicine, Amsterdam Research Institute Reproduction and Development, Academic Medical Centre, University of Amsterdam, Meibergdreef 9, 1105 AZ Amsterdam, the Netherlands
*Correspondence: danielm@imcb.a-star.edu.sg
https://doi.org/10.1016/j.stemcr.2020.03.013

SUMMARY

Spermatogenesis relies on exquisite stem cell homeostasis, the carefully balanced self-renewal and differentiation of spermatogonial stem cells (SSCs). Disturbing this equilibrium will likely manifest through sub- or infertility, a global health issue with often idiopathic presentation. In this respect, disease phenotypes caused by haploinsufficiency of otherwise vital developmental genes are of particular interest. Here, we show that mice heterozygous for *Trim28*, an essential epigenetic regulator, suffer gradual testicular degeneration. Contrary to previous reports we detect *Trim28* expression in spermatogonia, albeit at low levels. Further reduction through *Trim28* heterozygosity increases the propensity of SSCs to differentiate at the cost of self-renewal.

INTRODUCTION

Haploinsufficiency—a mechanism by which a loss-of-function mutation leaves only one functional copy of a gene and so leads to insufficient gene product (Deutschbauer et al., 2005)—is a major cause for developmental disorders (Kurotaki et al., 2002; Meechan et al., 2007), including male infertility (Chohan et al., 2018; Zhang et al., 2004). Haploinsufficiency phenotypes typically involve key genes that encode transcription factors and/or function in developmental processes (Dang et al., 2008; Deutschbauer et al., 2005). Unsurprisingly, several epigenetic factors with roles in development (NSD1, SUZ12, TRIM28, and many others) have been described to display haploinsufficiency phenotypes (Dalgaard et al., 2016; Kurotaki et al., 2002; Miro et al., 2009). TRIM28 (tripartite motif-containing protein 28), also known as KAP1, is an important mediator of epigenetic modifications. It is developmentally indispensable, as evident in the embryonic lethality of zygotic (Cammas et al., 2000) and maternal TRIM28 knockout mice (Lorthongpanich et al., 2013; Messerschmidt et al., 2012; Sampath Kumar et al., 2017), as well as the acute mortality of adult mice upon induction of systemic TRIM28 deletion (our unpublished data). Functionally, TRIM28 has been implicated in an array of processes, such as cell differentiation (Oleksiewicz et al., 2017), DNA damage response (White et al., 2006), silencing of retroviral elements (Rowe et al., 2010; Wolf et al., 2015), and epigenetic germline-to-soma inheritance (Lorthongpanich et al., 2013; Messerschmidt et al., 2012; Sampath Kumar et al., 2017; Seah and Messerschmidt, 2018). Haploinsufficiency of TRIM28 was shown to trigger a bistable obesity phenotype in mice, which was attributed to non-classical imprinted gene dysregulation (Dalgaard et al., 2016). Here, we report a new facet to TRIM28 haploinsufficiency, a remarkably penetrant infertility phenotype in heterozygous *Trim28* (*Trim28^{Het}*) male mice.

In the seminiferous tubules of the testis, millions of spermatozoa are produced on a daily basis through a process termed spermatogenesis (Johnson et al., 1980). During this process, spermatogonial stem cells (SSCs) undergo proliferative mitotic divisions at the basement membrane of seminiferous tubules to self-renew or form chains of spermatogonia that gradually differentiate into spermatocytes. Spermatocytes undergo reductive meiotic divisions to form haploid spermatids that are morphologically transformed into spermatozoa and eventually released into the lumen of the tubule. At present, TRIM28 has been shown to be expressed in spermatocytes and round spermatids. It is required for normal spermatogenesis yet the mechanism of the testicular degeneration upon TRIM28 deletion remains unclear and a possibly paracrine, non-cell-autonomous model has been evoked (Weber et al., 2002).

In this study, we uncovered and characterized a *Trim28* haploinsufficiency-induced infertility phenotype using conditional, constitutive, and inducible genetic approaches. We redefined the expression domain of TRIM28 in spermatogenesis and revealed a new, essential role of TRIM28 in the maintenance of the SSC compartment.

RESULTS

Trim28 Heterozygosity Causes Testicular Degeneration and Infertility

We observed a dramatic age-dependent decrease of fertility in heterozygous *Trim28* (*Trim28^{Het}*) males in our colony (Figures 1 and S1). At 32 weeks, testes of *Trim28^{Het}* mice were significantly smaller than those of age-matched

 © 2020 The Author(s).
This is an open access article under the CC BY license (http://creativecommons.org/licenses/by/4.0/).

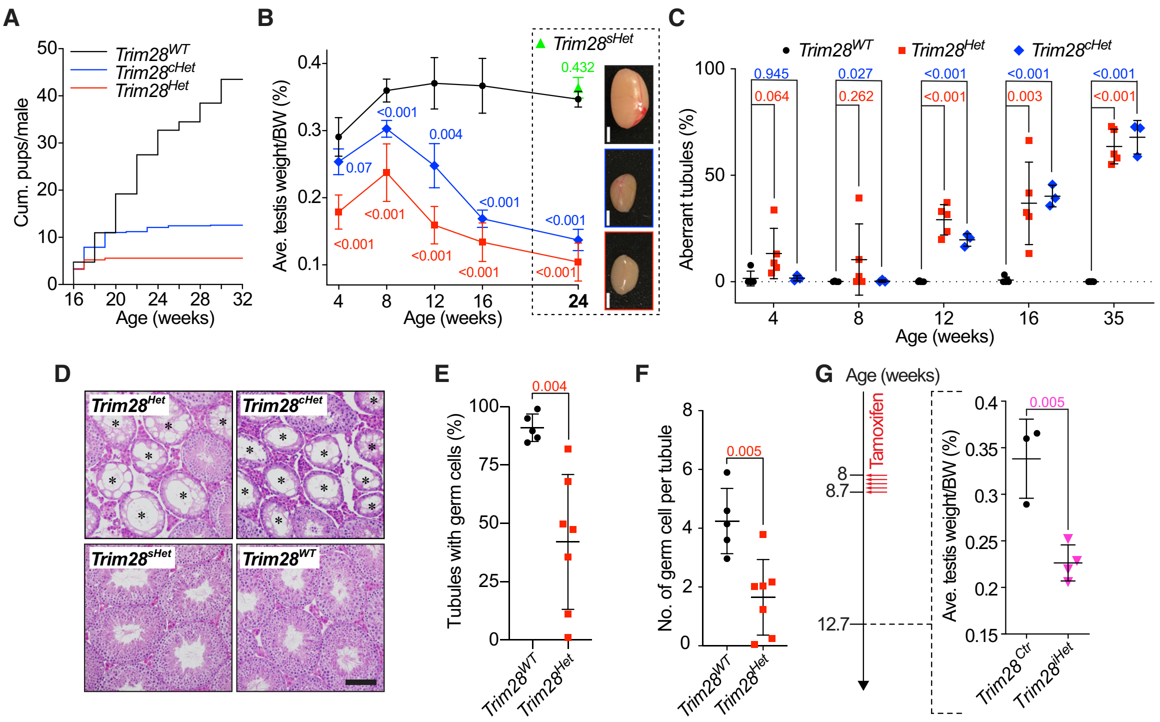

Figure 1. *Trim28* Haploinsufficiency in Germ Cells Leads to Testicular Degeneration and Premature Infertility

(A) Mating experiment demonstrating reduced fertility in *Trim28^{Het}* and *Trim28^{cHet}* males. n ≥ 3.

(B) Changes in testis size with age. Size of various heterozygous testes at 24 weeks (boxed). SD and p values are indicated; n ≥ 3. Diagrams show 24-week *Trim28^{WT}* (black box), *Trim28^{cHet}* (blue box), and *Trim28^{Het}* (red box) testes. Scale bars, 2 mm.

(C) Changes in percentage of aberrant seminiferous tubules with age. "Aberrant" refers to tubules that are largely depleted of germ cells. SD and p values are indicated; n ≥ 3.

(D) H&E staining of seminiferous tubules in 24-week testes. (*) denotes aberrant tubules. Scale bar, 50 μm.

(E and F) Percentage of germ cell-containing tubules (E) and germ cell count per tubule (F) in E18.5 embryonic testes. SD and p values are indicated; n ≥ 3.

(G) Testis size of *Trim28^{Ctr}* and *Trim28^{iHet}* males 4 weeks after tamoxifen injection. SD and p values are indicated; n ≥ 3.

Each symbol in (C), (E), (F), and (G) represents one mouse. Student's t test was used for all significance tests. See also Figure S1.

control mice (0.09% ± 0.01% vs 0.36% ± 0.02% of body weight [BW]) with their histology revealing numerous Sertoli cell-only tubules (Figure S1A). These animals also had smaller epididymides (0.09% ± 0.01% vs 0.14% ± 0.01% BW) that were largely devoid of spermatozoa (Figure S1B). The observed infertility could thus result from a loss of germ cells, which is supported by reduced cell counts at 16 weeks (Figures S1C–S1E). In addition, we observed the presence of abnormal spermatozoa, which may contribute, to a minor extent, to reduced fertility in younger mice (Figure S1F). Of note, in our hands *Trim28* haploinsufficiency did not induce an obesity phenotype (Figure S1G) as reported previously (Dalgaard et al., 2016; Whitelaw et al., 2010).

We conducted timed mating experiments to quantify the cumulative progeny of males and found that *Trim28^{Het}* males reproducibly become sterile as early as 20 weeks (Figure 1A). To test if the observed haploinsufficiency effects were germ cell-autonomous or related to systemic TRIM28 reduction, we produced germ cell-specific (conditional) *Trim28* heterozygous (*Trim28^{cHet}*) mice. In these animals, one *Trim28* allele is excised only in germ cells shortly before birth using the *Mvh-cre* deleter strain (Gallardo et al., 2007) (see Figure S2 for mouse model details). Although *Trim28^{cHet}* males sired more pups than *Trim28^{Het}* males initially, they also displayed an age-dependent decrease of fertility culminating in complete infertility (Figure 1A). For both *Trim28^{cHet}* and *Trim28^{Het}* males, the decreased fertility over time paralleled a gradual degeneration of the testis—evident in decreasing testis/BW ratio (Figures 1B and S1H) accompanied by an age-dependent increase in aberrant seminiferous tubules (Figures 1C and S1I). Sertoli cell-specific heterozygosity of *Trim28* (*Trim28^{sHet}*), induced through *Amh-cre* deletion (Holdcraft and Braun, 2004) (Figure S2), did not impact on testis size or tubule composition (Figures 1B and 1D). Despite the phenocopy of *Trim28^{Het}*

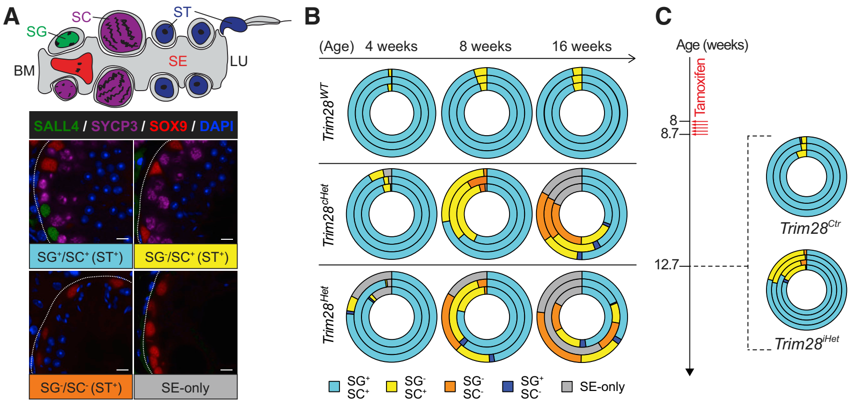

Figure 2. TRIM28 Deficiency Causes Sequential Loss of Germ Cells Beginning with the Spermatogonia

(A) Illustration of a seminiferous tubule segment showing spermatogonium (SG), spermatocytes (SC), spermatids (ST), and Sertoli cell (SE) (top panel). Four commonly observed compositions of germ cells in *Trim28^cHet^* and *Trim28^Het^* tubules from 4 to 16 weeks (bottom panel). Spermatogonia (SALL4, green), spermatocytes (SYCP3, purple), spermatids (DAPI morphology, blue), and Sertoli cells (SOX9, red). Dotted lines demarcate seminiferous tubules. Scale bars, 10 μm.

(B) Donut chart tracing the shift in germ cell composition of tubules with age.

(C) Shift in germ cell composition of tubules in *Trim28^iHet^* testes 4 weeks after tamoxifen injection. Each concentric circle represents analysis of one mouse.

See also Figure S3.

animals, we noticed a discrepancy in the testicular phenotype of *Trim28^{cHet}* mice at 4 weeks (Figures 1B and 1C), which may be explained by early prenatal effects of TRIM28 reduction (in *Trim28^{Het}*) resulting in less pre-spermatogonia just before birth (E18.5) (Figures 1E and 1F).

Finally, we used a tamoxifen-inducible, germ cell-specific *Trim28*-heterozygous mouse model (*Trim28^{iHet}*) based on the *Mvh-cre^{ERT2}* (John et al., 2008) (Figure S2) to test if the degenerative phenotype was dependent on pre- or postnatal deletion of one *Trim28* allele. Eight-week-old males were injected with tamoxifen, and testes of *Trim28^{iHet}* males were found to be much smaller than those of *Trim28^{Ctr}* mice (0.23% ± 0.02% vs 0.34% ± 0.04% BW) 4 weeks later (Figure 1G). This suggests that testicular degeneration was independent of prenatal TRIM28 reduction. Altogether these findings demonstrate the germ cell-autonomous requirement of TRIM28 for normal spermatogenesis in young and adult male mice.

Testicular Degeneration Originates from the Spermatogonia Compartment

We established that *Trim28* haploinsufficiency causes a gradual, age-dependent testicular degeneration. Next, we surveyed the germ and somatic cell populations within the seminiferous tubules using their respective cellular markers—SALL4 (spermatogonia, SG), SYCP3 (spermatocytes, SC), and SOX9 (Sertoli cell, SE)—at various ages. This analysis revealed four types of tubules commonly observable in both heterozygous mutant models (*Trim28^{Het}* and *Trim28^{cHet}*): (1) normal tubules that contain SALL4/SYCP3-positive cells (SG^+/SC^+), (2) spermatogonia-deficient tubules containing SYCP3-positive but not SALL4-positive cells (SG^-/SC^+), (3) spermatogonia- and spermatocyte-deficient tubules without SALL4/SYCP3-positive cells (SG^-/SC^-), and (4) Sertoli cell-only tubules containing only SOX9-positive cells (SE-only) (Figure 2A). Note that, apart from SE-only, all tubules contained spermatids (ST), which are identifiable by DAPI staining. In control animals, up to 16 weeks, we found no SG^-/SC^- or SE-only tubules and rarely observed SG^-/SC^+ tubules (4.25% ± 0.79%) (Figure 2B, top row and Figure S3). Conversely, in *Trim28^{cHet}* testis, while showing a majority SG^+/SC^+ tubules at 4 weeks (94.30% ± 2.55%), the proportion of SG^-/SC^+ tubules increased dramatically (31.08% ± 6.67%) at 8 weeks (with 64.26% ± 7.55% remaining SG^+/SC^+). This phenotype was exacerbated with the appearance of SG^-/SC^- (22.26% ± 7.21%) and SE-only (17.90% ± 1.03%) tubules, further reducing SG^+/SC^+ (40.78% ± 9.75%) tubules at 16 weeks (Figure 2B, middle row and Figure S3). *Trim28^{Het}* testis showed a similar sequential degenerative trend with the appearance of first SG^-/SC^+ followed by SG^-/SC^- and finally SE-only tubules. However, the defects were more pronounced and yet more variable with pre-existing SE-only tubules at 4 weeks (10.31% ± 7.72%) (Figure 2B, bottom row and Figure S3). This is again likely due to prenatal effects, consistent with our previous observation (Figure 1E). We also observed, in both mutant models but not controls, the sporadic appearance of spermatocyte-deficient tubules (SG^+/SC^-, up to 3.3% at 16 weeks) (Figures 2B and S3). Because these tubules contain spermatids, they are probably caused by shedding of spermatocytes from the seminiferous epithelium.

These observations reveal a sequential disappearance of first spermatogonia, then spermatocytes, and finally spermatids, pointing toward a spermatogonia (possibly SSC) defect (Figure 2B). Importantly, spermatogonia loss was also recapitulated in the *Trim28^{iHet}* model when analyzed 4 weeks after tamoxifen injection (17.61% ± 3.45%

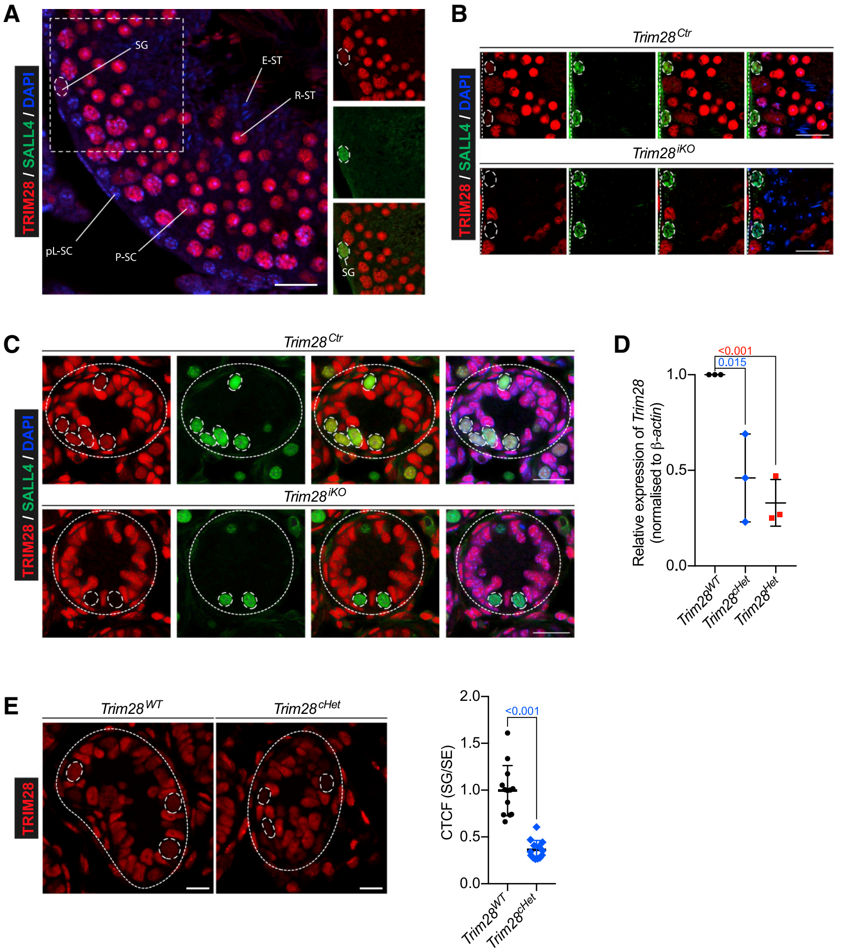

Figure 3. TRIM28 Is Expressed in Spermatogonia but at a Lower Level Compared with Later Germ Cells

(A) Immunofluorescence staining of adult testis indicating TRIM28 expression (red) in germ cells. Scale bar, 20 μm. Seminiferous tubule segment focusing on TRIM28 (red) expression in spermatogonium (green) (small panels). SG, spermatogonium; pL-SC, preleptotene spermatocyte; P-SC, pachytene spermatocyte; R-ST, round spermatid; E-ST, elongated spermatid.

(B and C) Immunofluorescence staining of adult (B) and P7 (C) *Trim28^{Ctr}* and *Trim28^{iKO}* testes showing expression and absence of TRIM28 in wild-type and knockout SALL4-positive spermatogonia, respectively. Seminiferous tubules are demarcated by dotted lines. Scale bars, 20 μm.

(D) *Trim28* expression in magnetic-activated cell sorting-sorted THY1-enriched spermatogonia. Each symbol represents one biological replicate. SD and p values are indicated; n = 3.

(E) Immunofluorescence staining of P7 *Trim28^{WT}* and *Trim28^{cHet}* testes showing expression and reduction of TRIM28 in SALL4-positive spermatogonia. Scale bars, 10 μm. Ratio of SG CTCF to SE CTCF based on measurement of fluorescence intensity. SE, Sertoli cell; CTCF, corrected total cell fluorescence. Each symbol represents one cell. SD and p values are indicated; n ≥ 10. Student's t test was used for all significance tests.

See also Figure S4.

SG^{-}/SC^{+}, 80.52% ± 2.27% SG^{+}/SC^{+}) (Figures 2C and S3). Taken together, our findings suggest that testicular degeneration is primarily driven by the depletion of spermatogonia, which is triggered by reduction of TRIM28.

Spermatogonia Express Low Levels of TRIM28

Expression of TRIM28 during spermatogenesis was reported in mid-pachytene spermatocytes to early elongating spermatids and not at earlier meiotic stages or in spermatogonia (Weber et al., 2002). This, in combination with our observed spermatogonia depletion, may suggest a non-cell-autonomous, perhaps paracrine, effect of TRIM28 in spermatocytes on spermatogonia, which is at odds with chemical methods for germ cell depletion and SSC-mediated regeneration (Wang et al., 2010). We therefore reassessed TRIM28 expression in the seminiferous tubule by immunofluorescence staining. We found TRIM28 to be widely expressed in both germ and somatic cells, including, spermatocytes, spermatids, and Sertoli cells, as previously described. However, we further detected TRIM28 in preleptotene spermatocytes and crucially also in SALL4-positive spermatogonia, although at a much lower intensity (Figures 3A and S4A). No staining was found in elongated spermatids or spermatozoa. Our findings were independent of fixation method and consistent with different TRIM28 antibodies (Figure S4B). To further confirm the specificity of the staining, we ablated TRIM28 in all germ cells using our *Mvh-cre^{ERT2}*-inducible, germ cell-specific *Trim28*-knockout mouse model (*Trim28^{iKO}*). Although in adult control (*Trim28^{Ctr}*) animals SALL4-positive spermatogonia continued faint TRIM28 staining upon induction, adult *Trim28^{iKO}* SALL4-positive spermatogonia lost all TRIM28 staining post-recombination (Figure 3B). This was also recapitulated in seminiferous tubules of postnatal day 7 (P7) pup testes, which harbor only spermatogonia (of diverse differentiation stages) and Sertoli cells (Figure 3C). The specific protein expression of TRIM28 in spermatogonia is further supported by multiple recent RNA sequencing (RNA-seq) datasets (Hammoud et al., 2015; Helsel et al., 2017). *Trim28* was detectable in SSCs (ID4-eGFP bright cells) as well as progenitor undifferentiated spermatogonia (ID4-eGFP dim cells) at the transcript level (Helsel et al., 2017) (Figure S4C). Indeed, in THY1-enriched

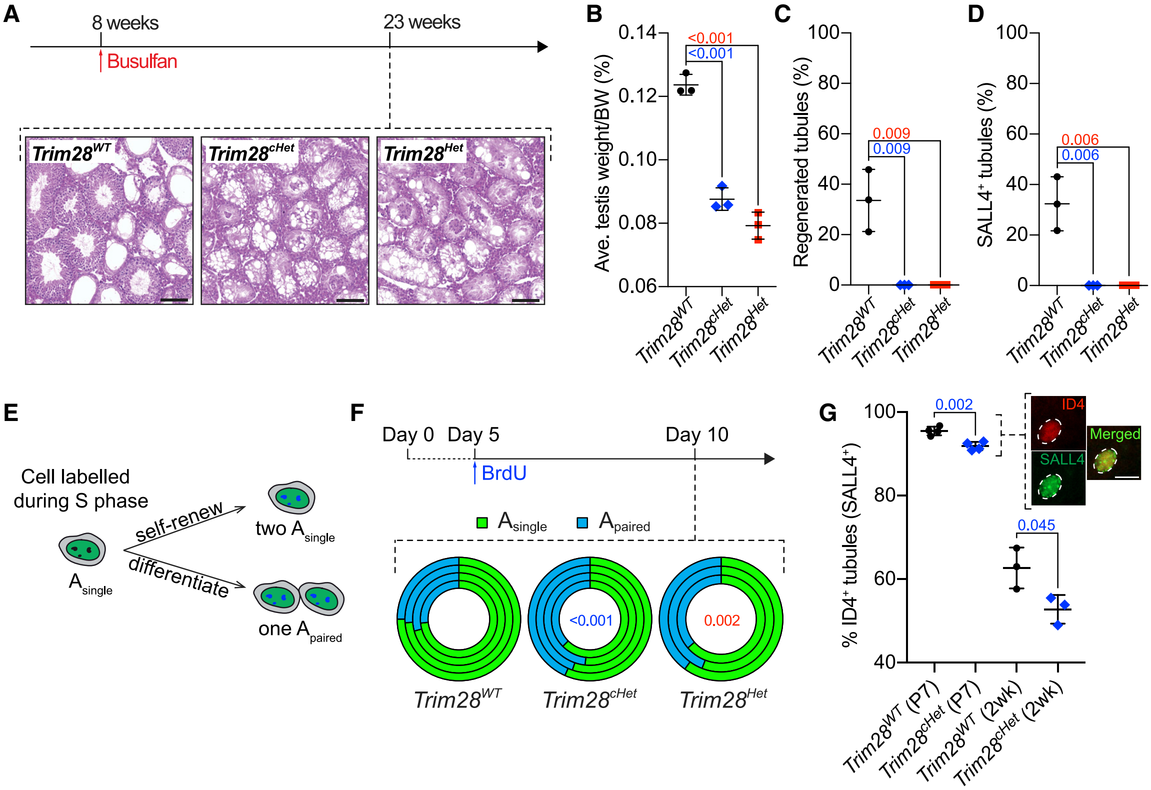

Figure 4. SSC Requires TRIM28 to Maintain Spermatogenesis

(A) Histology of testes 15 weeks after busulfan injection. Scale bars, 50 μm.

(B) Testis size 15 weeks after busulfan injection. SD and p values are indicated; $n = 3$.

(C) Percentage of regenerated tubules. SD and p values are indicated; $n = 3$.

(D) Percentage of SALL4-positive tubules. SD and p values are indicated; $n = 3$.

(E) BrdU tracing experiment. A $BrdU^+$ A_{single} spermatogonium (putative SSC), upon cell division, either undergoes (the "event") cytokinesis forming two A_{single} or two attached A_{paired} spermatogonia, indicating a tendency toward self-renewal or differentiation, respectively.

(F) BrdU experiment scheme and donut chart showing percentage of BrdU-positive events (either self-renewal or differentiation) in whole-mount seminiferous tubules 5 days after BrdU injection. Each concentric circle represents analysis of one mouse and the numbers indicate p values with respect to *Trim28*^{WT}. See also Figure S5.

(G) Percentage of ID4-positive (and SALL4-positive) tubules in P7 and 2-week testes. SD and p values are indicated; $n \geq 3$. Validation of ID4 immunostaining on a P7 SALL4-positive spermatogonium. Scale bar, 10 μm.

Each symbol in (B), (C), (D), and (G) represents one mouse. Student's t test was used for all significance tests.

P7 germ cells, we detected *Trim28* by qPCR and saw approximately 50% reduction in *Trim28*^{Het} and *Trim28*^{cHet} mutants (Figure 3D), which translated to a similar decrease in protein level in heterozygous spermatogonia (Figure 3E). In addition to mouse testis, we stained adult human testis for TRIM28 and found a highly conserved expression pattern from mice to men, including in spermatogonia (Figure S4D).

Having shown and validated the expression of TRIM28 in spermatogonia, in light of the progressive loss of spermatogonia, we hypothesize that, without sufficient intrinsic TRIM28, SSCs are unable to maintain spermatogenesis.

SSCs Require TRIM28 to Maintain Spermatogenesis

After the initial wave(s), subsequent rounds of spermatogenesis initiate from the SSC pool, which is sustained by self-renewal division (Law et al., 2019; Yoshida et al., 2006). To determine the ability of TRIM28-deficient SSCs to maintain spermatogenesis, we depleted adult mouse testes of germ cells by administering a single intraperitoneal dose of busulfan (30 mg/kg), a DNA-alkylating agent that targets and eliminates proliferating cells, such as spermatogonia (Wang et al., 2010), and assessed the repopulation of these testes 15 weeks after injection (Figure 4A). Although *Trim28*^{WT} testes regenerated tubules to some

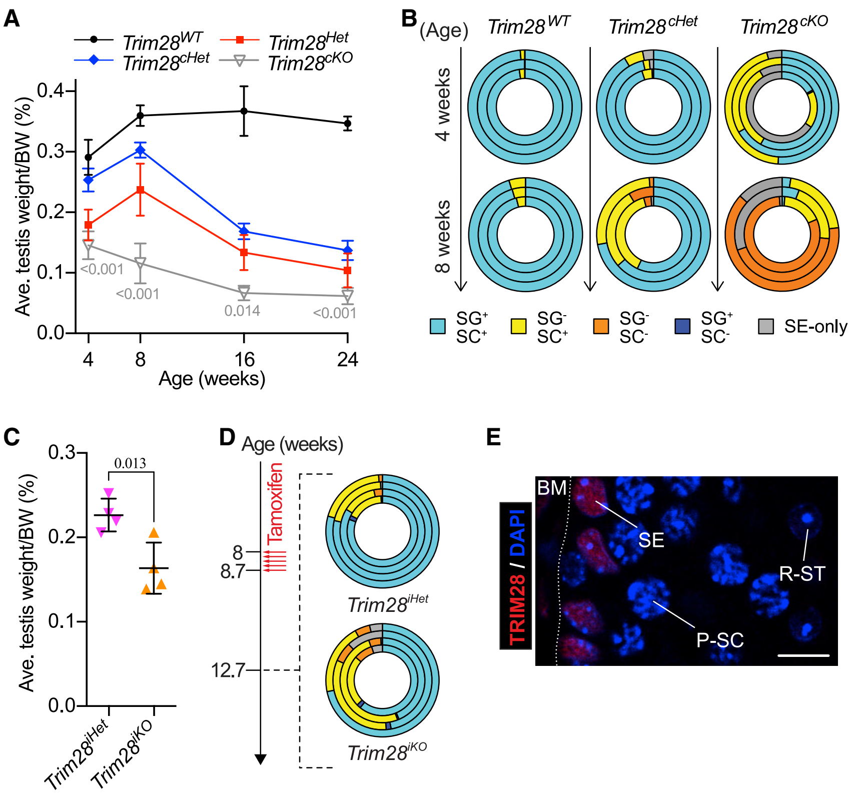

Figure 5. TRIM28 KO Worsens Phenotype but Reveals Dispensability in Germ Cell Differentiation

(A) Changes in testis size with age. SD and p values are indicated; n ≥ 3.

(B) Shift in germ cell composition of seminiferous tubules with age.

(C) Testis size of *Trim28^{iHet}* and *Trim28^{iKO}* males 4 weeks after tamoxifen injection. SD and p values are indicated; n = 4.

(D) Shift in germ cell composition of seminiferous tubules in *Trim28^{iHet}* and *Trim28^{iKO}* testes 4 weeks after tamoxifen injection.

(E) Immunofluorescence staining of a *Trim28^{iKO}* seminiferous tubule at 4 weeks (P1–P3 tamoxifen injection) showing absence of TRIM28 in pachytene spermatocytes (P-SC) and round spermatids (R-ST). Scale bar, 10 μm. BM, basement membrane.

Each concentric circle in (B) and (D) represents analysis of one mouse. See also Figure S3. Student's t test was used for all significance tests.

extent, neither of the heterozygote models showed any recovery (Figures 4A–4C). The proportion of regenerated tubules in *Trim28^{WT}* testes (33.61% ± 12.32%) was correlated to the proportion of SALL4-positive tubules (32.39% ± 10.66%), indicating that the replenished germ cells were derived from SSCs as expected (Figures 4C and 4D). The lack of spermatogonia, and therefore regenerated tubules, in the *Trim28^{Het}* and *Trim28^{cHet}* testes hence implies SSCs exhaustion.

We next investigated the dynamics of the *Trim28*-heterozygous SSCs. According to the recent "Revised A_{single} model" for SSC self-renewal, A_{single} spermatogonia that express high levels of ID4 are the SSCs (Lord and Oatley, 2017). As spermatogonia proliferate, the increase in spermatogonial chain length (i.e., A_{paired}, $A_{aligned}$) directly correlates with their propensity toward a more differentiated state (Lord and Oatley, 2017). With this in mind, we administered bromodeoxyuridine (BrdU) to P5 pups and traced the fate of BrdU-incorporated A_{single} spermatogonia after 5 days since cell division occurs every 3 to 4 days (de Rooij, 2017). Using GFRα1, an early undifferentiated spermatogonia marker that includes SSCs (Nakagawa et al., 2010) in a whole-mount immunofluorescence staining of seminiferous tubules, we scored divisions forming either two detached A_{single} cells (self-renewal) or an A_{paired} cell pair (differentiation) (Figure 4E). Comparing *Trim28^{WT}* (73.27% ± 1.77%) with *Trim28^{cHet}* (56.55% ± 4.48%) and *Trim28^{Het}* (59.84% ± 3.88%) "A_{single}" events, TRIM28-deficient SSCs showed a propensity to form A_{paired} spermatogonia and hence rather differentiate than self-renew (Figure 4F), thereby likely resulting in premature depletion of the stem cell pool. In support of this hypothesis, we saw a significant and progressive reduction in the proportion of ID4-positive tubules in *Trim28^{cHet}* compared with *Trim28^{WT}* testes at P7 (91.91% ± 1.01% vs 95.49% ± 1.02%) and 2 weeks (52.82% ± 3.41% vs 62.73% ± 4.89%) (Figure 4G). Hence, we show that the loss-of-fertility phenotype in the *Trim28^{Het}* males likely succeeds the exhaustion of the SSC pool due to their propensity toward differentiation.

Full TRIM28 Loss Worsens Phenotype yet Reveals Dispensability for Germ Cell Differentiation

Having characterized the phenotype in the heterozygotes, we proceeded to address the obvious question: What ensues from the complete knockout of TRIM28 in germ cells? Apart from our *Trim28^{iKO}* model, we generated a germ cell-specific (conditional) *Trim28* knockout mouse (*Trim28^{cKO}*) using the *Mvh-Cre* deleter strain to study the progression of TRIM28-null germ cells into adulthood.

Complete ablation of TRIM28 in all germ cells (on a heterozygous background) resulted in accelerated

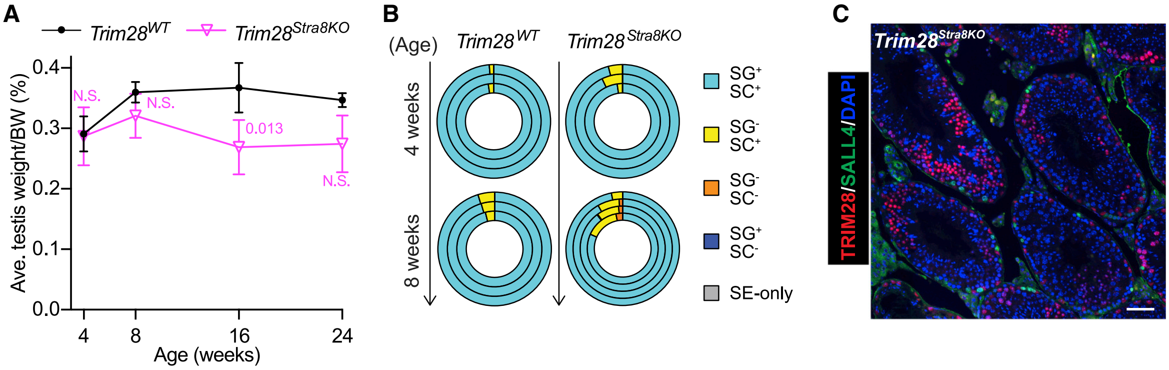

Figure 6. *Trim28^{Stra8KO}* Mice Undergo Less Severe Testicular Degeneration

(A) Changes in testis size with age. SD and p values are indicated; $n \geq 3$.

(B) Shift in germ cell composition of seminiferous tubules with age. Each concentric circle represents analysis of one mouse. See also Figure S3.

(C) Immunofluorescence staining of a 62-week-old *Trim28^{Stra8KO}* testis. Scale bar, 50 μm.

degeneration in terms of testis size (Figure 5A) as well as tubule composition where *Trim28^{cKO}* testes had started losing half their SG$^+$/SC$^+$ tubules at 4 weeks (48.23% ± 21.84%) and almost all of the rest at 8 weeks (3.17% ± 2.44% SG$^+$/SC$^+$) (Figure 5B). This dosage phenomenon is independent of pre- or postnatal deletion of TRIM28 since removing both alleles of *Trim28* in adult germ cells furthered progression (compared with *Trim28^{iHet}*) of testis size reduction (0.16% ± 0.03% vs 0.23% ± 0.02% BW) and spermatogonia loss (31.81% ± 13.60% SG$^-$/SC$^+$ vs 17.61% ± 3.45% SG$^-$/SC$^+$) (Figures 5C and 5D). Another striking observation was the presence of TRIM28-negative spermatocytes and spermatids (Figure 5E), which indicates that TRIM28-null spermatogonia were able to differentiate into later germ cells instead of being lost to cell death. We can thus posit that TRIM28 functions to maintain the SSC pool but, beyond that, is not necessary for germ cell differentiation.

Interestingly, testes of conditional knockout mice generated using the *Stra8-Cre* deleter strain (*Trim28^{Stra8KO}*) (Sadate-Ngatchou et al., 2008) did not appear to be significantly affected by the lack of TRIM28 (Figure 6A). This was mirrored in the tubule composition of *Trim28^{Stra8KO}* testes, which were phenotypically similar to the wild type at 4 weeks (95.53% ± 2.45% SG$^+$/SC$^+$) and 8 weeks (89.98% ± 6.29% SG$^+$/SC$^+$) (Figures 6B and S3). In fact, seminiferous tubules of *Trim28^{Stra8KO}* testes still harbored various TRIM28-negative germ cell layers (including SALL4-positive spermatogonia) at 62 weeks (more than thrice the time *Trim28^{Het}* and *Trim28^{cHet}* took to become infertile) (Figure 6C). Although (in)efficiency of Cre-mediated recombination might render such a milder phenotype, another logical explanation could be the activation of the *Stra8-cre* in late undifferentiated spermatogonia (in response to retinoic acid) just before transiting into differentiated spermatogonia (Anderson et al., 2008). This would concur with our finding that SSCs require TRIM28 since SSCs in these mice should express wild-type levels of TRIM28.

DISCUSSION

Ablation of key developmental genes has been shown to be incompatible with life, but the haploinsufficient effects of such genes are relatively poorly understood. Our study on TRIM28 reveals that heterozygous mice are viable but exhibit a premature infertility phenotype in male animals. Characterization of this haploinsufficiency phenotype using different mouse models specifies the spatial (as opposed to temporal) importance of TRIM28 function within spermatogonia and provides a new insight into the role of this developmentally essential regulator in spermatogenesis, namely the cell-autonomous homeostasis of the SSC compartment. This expands on a previous study that suggests, on the basis of restricted detection of TRIM28 in mid-pachytene spermatocytes to early elongating spermatids, a TRIM28 requirement for spermatogonia maintenance through a paracrine feedback mechanism from Sertoli cells, or meiotic or post-meiotic germ cells (Weber et al., 2002). Conversely, we irrefutably demonstrated the expression (and reduction due to heterozygosity) of TRIM28 in spermatogonia at RNA and protein levels, making a cell-autonomous effect most plausible. Our finding of TRIM28 expression in spermatogonia and presumably SSC is also supported by RNA-seq datasets of ID4-eGFP bright (and dim) spermatogonia (Helsel et al., 2017) as well as THY1-, OCT4-, and KIT-positive spermatogonia (Hammoud et al., 2015). Interestingly, the expression levels of

TRIM28 in spermatogonia are extraordinarily low compared with later spermatocytes and spermatids, which probably confounded previous studies. We speculate that it is this low-level expression that makes these cells particularly vulnerable to haploinsufficiency. This, together with the spermatogonia-initiated germ cell depletion and poor regeneration of chemically depleted testes in *Trim28*^{cHet} mice, and propensity of TRIM28-deficient SSC to differentiate, clearly point to a previously unknown role of TRIM28 in SSC.

We showed that complete loss of TRIM28 in spermatogonia potentiated the haploinsufficiency phenotype, presenting a dosage sensitivity phenomenon of TRIM28 while suggesting that other mechanisms of spermatogonia depletion could be underway. Our knockout mouse models also highlighted the dispensability of TRIM28 for germ cell differentiation from spermatogonia to spermatozoa, which is interesting considering that TRIM28 is an important epigenetic regulator and aberrant epigenetic modifications during meiosis have been found to cause meiotic defects (Zamudio et al., 2008). Of note, *Trim28* deletion using the *Stra8-Cre* deleter strain yielded an initially unexpected milder testicular degeneration phenotype, which could be explained by the activation of *Stra8-cre* in response to retinoic acid in late undifferentiated spermatogonia (Anderson et al., 2008), hence avoiding recombination in SSC. Although this supports our hypothesis of the role TRIM28 has in SSC, it reveals the utility of this model for the study of early progenitor spermatogonia and SSC.

Trim28 haploinsufficiency has previously been shown to trigger obesity (Dalgaard et al., 2016), which is positively correlated with male infertility (Kasturi et al., 2008). However, neither were *Trim28*-heterozygous mice in our study presented with obesity nor were such obese mice reported to have impaired fertility. This discrepancy may be explained by the divergent genetic background of animals used, since our *Trim28* colony is of pure C57BL/6J background (Messerschmidt et al., 2012), while other studies reporting bistable obesity, yet not infertility, were carried out on an FVB/NJ background (Dalgaard et al., 2016; Whitelaw et al., 2010). Interestingly, this also implies that the observed infertility is not obesity-related but germ cell-autonomous as further supported by germ cell-specific TRIM28 haploinsufficiency.

Finally, the adult haploinsufficiency phenotypes of developmentally essential factors are of particular interest in the study of human syndromes. We show that TRIM28 is also expressed in human spermatogonia. Moreover, RNA-seq analysis of human testis samples revealed a broad range of *Trim28* expression levels (82.1–210.8 reads per kilobase of transcript per million mapped reads) (Uhlen et al., 2015), possibly hinting toward a relevance of TRIM28 insufficiency in idiopathic infertility of men.

EXPERIMENTAL PROCEDURES

Mouse Models

All animal work was performed under and in accordance with the approved IACUC protocol (no. 181393). Derivation of *Trim28* floxed (*Trim28*$^{f/f}$) mouse was reported previously (Cammas et al., 2000). *Mvh-Cre* (Gallardo et al., 2007), *Amh-Cre* (Holdcraft and Braun, 2004), *Mvh-Cre*^{ERT2} (John et al., 2008), and *Stra8-Cre* (Sadate-Ngatchou et al., 2008) transgenic mice were obtained from The Jackson Laboratory and used as described. For inducible models, adult mice were injected intraperitoneally (i.p.) with 2 mg tamoxifen (Sigma) for 5 consecutive days. For neonates, tamoxifen was injected in increasing doses (50, 75, and 100 μg) for 3 consecutive days starting at P1.

Mating Experiment

Males were mated with *Trim28*^{WT} females. Vaginal plug was checked daily and pups were counted at birth. Total number of pups from males of the same genotype was tallied at each stipulated time frame and divided by the number of successful vaginal plugs to obtain the average litter size for each successful mating by a male.

Histology

Testes and epididymides were fixed overnight at 4°C in Bouin solution (Sigma) or modified Davidson's fixative (mDF) (30% formaldehyde, 15% ethanol, 5% glacial acetic acid, 50% water) and embedded in paraffin. Testes of embryonic day 18.5 (E18.5) embryos were fixed in mDF for 5 h. Quantification of Sertoli cell-only tubules on hematoxylin and eosin (H&E)-stained slides involved three sections (>200 μm apart) from each testis. For immunofluorescence staining, heat-induced epitope retrieval was done in 10 mM sodium citrate buffer (pH 6; 105°C for 25 min). Sections were blocked (10% BSA, 3% milk, 0.1% Triton X-100 in PBS) for 1 h at room temperature (RT), incubated with primary antibodies at 37°C for 1 h, before secondary antibody incubation for 1 h at RT. Coverslip was mounted with Hydromount (National Diagnostics) containing 1 μg/mL DAPI (Sigma). Counting of tubules (and cells) was done manually and all tubules within each testis section were counted. See Table S2 for antibodies used. Corrected total cell fluorescence was derived as described (El-Sharkawey, 2016), parameters for calculation were measured using Fiji (Schindelin et al., 2012).

Spermatogonia Isolation and Cell Sorting

Spermatogonia isolation was performed on neonate testes (P6–P8) as described previously (Goodyear and Brinster, 2017). Magnetic-activated cell sorting of THY1-positive cells was done as per manufacturer's instructions (Miltenyi Biotec).

RNA Isolation and Reverse Transcription Quantitative PCR

RNA from spermatogonia was isolated using the Arcturus PicoPure RNA Isolation Kit (Thermo Fisher Scientific) following the manufacturer's instructions. Total RNA was converted using the High-

Capacity cDNA Reverse Transcription Kit (Thermo Fisher Scientific). qPCR assays were designed with the Universal Probe Library Assay Design Center (Roche), and performed (three technical replicates) on the CFX96 Touch Real-Time PCR Detection System (Bio-Rad). Target genes were normalized to *β-actin* and their relative expression analyzed using the $2^{-\Delta\Delta ct}$ method. See Table S1 for primers and probes used.

Testicular Germ Cell Depletion and Regeneration

Busulfan (Sigma) was prepared by dissolving in DMSO (Sigma) and diluting with an equal volume of water. A single dose (30 mg/kg) was administered via i.p. injection. Percentage of regenerated seminiferous tubules (i.e., with germ cells up to the spermatid stage) was quantified on H&E-stained testis sections.

BrdU Tracing Experiment (Seminiferous Tubule Whole-Mount Staining)

P5 neonates were given 10 μL of Amersham Cell Proliferation Labeling Reagent (GE Healthcare) per gram of body weight via i.p. injection and testes were isolated 5 days later. The tunica albuginea of the testis was removed, and seminiferous tubules were mechanically separated and fixed in 4% paraformaldehyde (Sigma) for 5 h at 4°C. Fixed tubules were denatured with 3.5 M HCl for 2 min and blocked (2% BSA, 10% fetal bovine serum, 0.3% Triton X-100 in PBS) for 4 h at RT. Tubules were incubated with primary antibodies overnight at 4°C followed by secondary antibodies for 1.5 h at RT. Coverslip was mounted using DAPI-containing Hydromount. At least 1 cm of tubule was analyzed. A_{single} cells were cells that stood alone or if cytoplasmic connections were present, cells that were more than one nuclear length apart. An A_{single} event was counted when an A_{single} cell was spotted or when two A_{single} cells were found in close proximity (within the counting frame). A_{paired} cells were defined as two cells that were connected and within one nuclear length from each other. See Table S2 for antibodies used.

Statistical Analysis

Data are represented as mean ± SD and were analyzed using Student's t test (p values as indicated). N.S. denotes not significant ($p > 0.05$). At least three biological replicates were used for each experiment.

SUPPLEMENTAL INFORMATION

Supplemental Information can be found online at https://doi.org/10.1016/j.stemcr.2020.03.013.

AUTHOR CONTRIBUTIONS

J.H.L.T. conceived and performed the experiments, analyzed the data, and wrote the manuscript. H.W. performed the experiments and analyzed the data. A.M.M.v.P. contributed reagents, supervised analysis, and contributed to the manuscript. P.K. conceived and supervised the experiments. D.M.M. conceived and supervised the study, conceived and performed the experiments, analyzed the data, and wrote the manuscript.

ACKNOWLEDGMENTS

We thank the Messerschmidt, Kaldis, and Guccione lab for support and scientific discussions. SIgN Flow Cytometry Services for FACS sorting of cells, and Lim Shu Ly for discussion and advice. This study was funded by National Research Foundation Singapore, NRF-NRFF2015-05.

Received: January 8, 2020
Revised: March 13, 2020
Accepted: March 17, 2020
Published: April 16, 2020

REFERENCES

Anderson, E.L., Baltus, A.E., Roepers-Gajadien, H.L., Hassold, T.J., de Rooij, D.G., van Pelt, A.M., and Page, D.C. (2008). Stra8 and its inducer, retinoic acid, regulate meiotic initiation in both spermatogenesis and oogenesis in mice. Proc. Natl. Acad. Sci. U S A *105*, 14976–14980.

Cammas, F., Mark, M., Dolle, P., Dierich, A., Chambon, P., and Losson, R. (2000). Mice lacking the transcriptional corepressor TIF1-beta are defective in early postimplantation development. Development *127*, 2955–2963.

Chohan, H., Esfandiarei, M., Arman, D., Van Raamsdonk, C.D., van Breemen, C., Friedman, J.M., and Jett, K.A. (2018). Neurofibromin haploinsufficiency results in altered spermatogenesis in a mouse model of neurofibromatosis type 1. PLoS One *13*, e0208835.

Dalgaard, K., Landgraf, K., Heyne, S., Lempradl, A., Longinotto, J., Gossens, K., Ruf, M., Orthofer, M., Strogantsev, R., Selvaraj, M., et al. (2016). Trim28 haploinsufficiency triggers bi-stable epigenetic obesity. Cell *164*, 353–364.

Dang, V.T., Kassahn, K.S., Marcos, A.E., and Ragan, M.A. (2008). Identification of human haploinsufficient genes and their genomic proximity to segmental duplications. Eur. J. Hum. Genet. *16*, 1350–1357.

de Rooij, D.G. (2017). The nature and dynamics of spermatogonial stem cells. Development *144*, 3022.

Deutschbauer, A.M., Jaramillo, D.F., Proctor, M., Kumm, J., Hillenmeyer, M.E., Davis, R.W., Nislow, C., and Giaever, G. (2005). Mechanisms of haploinsufficiency revealed by genome-wide profiling in yeast. Genetics *169*, 1915–1925.

El-Sharkawey, A. (2016). Calculate the Corrected Total Cell Fluorescence (CTCF).

Gallardo, T., Shirley, L., John, G.B., and Castrillon, D.H. (2007). Generation of a germ cell-specific mouse transgenic Cre line, Vasa-Cre. Genesis *45*, 413–417.

Goodyear, S., and Brinster, R. (2017). Isolation of the spermatogonial stem cell-containing fraction from testes. Cold Spring Harbor Protoc. *2017*. https://doi.org/10.1101/pdb.prot094185.

Hammoud, S.S., Low, D.H.P., Yi, C., Lee, C.L., Oatley, J.M., Payne, C.J., Carrell, D.T., Guccione, E., and Cairns, B.R. (2015). Transcription and imprinting dynamics in developing postnatal male germline stem cells. Genes Dev. *29*, 2312–2324.

Helsel, A.R., Yang, Q.E., Oatley, M.J., Lord, T., Sablitzky, F., and Oatley, J.M. (2017). ID4 levels dictate the stem cell state in mouse spermatogonia. Development *144*, 624–634.

Holdcraft, R.W., and Braun, R.E. (2004). Androgen receptor function is required in Sertoli cells for the terminal differentiation of haploid spermatids. Development *131*, 459–467.

John, G.B., Gallardo, T.D., Shirley, L.J., and Castrillon, D.H. (2008). Foxo3 is a PI3K-dependent molecular switch controlling the initiation of oocyte growth. Dev. Biol. *321*, 197–204.

Johnson, L., Petty, C.S., and Neaves, W.B. (1980). A comparative study of daily sperm production and testicular composition in humans and rats. Biol. Reprod. *22*, 1233–1243.

Kasturi, S.S., Tannir, J., and Brannigan, R.E. (2008). The metabolic syndrome and male infertility. J. Androl. *29*, 251–259.

Kurotaki, N., Imaizumi, K., Harada, N., Masuno, M., Kondoh, T., Nagai, T., Ohashi, H., Naritomi, K., Tsukahara, M., Makita, Y., et al. (2002). Haploinsufficiency of NSD1 causes Sotos syndrome. Nat. Genet. *30*, 365–366.

Law, N.C., Oatley, M.J., and Oatley, J.M. (2019). Developmental kinetics and transcriptome dynamics of stem cell specification in the spermatogenic lineage. Nat. Commun. *10*, 2787.

Lord, T., and Oatley, J.M. (2017). A revised Asingle model to explain stem cell dynamics in the mouse male germline. Reproduction *154*, R55–R64.

Lorthongpanich, C., Cheow, L.F., Balu, S., Quake, S.R., Knowles, B.B., Burkholder, W.F., Solter, D., and Messerschmidt, D.M. (2013). Single-cell DNA-methylation analysis reveals epigenetic chimerism in preimplantation embryos. Science *341*, 1110–1112.

Meechan, D.W., Maynard, T.M., Gopalakrishna, D., Wu, Y., and LaMantia, A.S. (2007). When half is not enough: gene expression and dosage in the 22q11 deletion syndrome. Gene Expr. *13*, 299–310.

Messerschmidt, D.M., de Vries, W., Ito, M., Solter, D., Ferguson-Smith, A., and Knowles, B.B. (2012). Trim28 is required for epigenetic stability during mouse oocyte to embryo transition. Science *335*, 1499–1502.

Miro, X., Zhou, X., Boretius, S., Michaelis, T., Kubisch, C., Alvarez-Bolado, G., and Gruss, P. (2009). Haploinsufficiency of the murine polycomb gene Suz12 results in diverse malformations of the brain and neural tube. Dis. Model. Mech. *2*, 412–418.

Nakagawa, T., Sharma, M., Nabeshima, Y.-i., Braun, R.E., and Yoshida, S. (2010). Functional hierarchy and reversibility within the murine spermatogenic stem cell compartment. Science *328*, 62–67.

Oleksiewicz, U., Gladych, M., Raman, A.T., Heyn, H., Mereu, E., Chlebanowska, P., Andrzejewska, A., Sozanska, B., Samant, N., Fak, K., et al. (2017). TRIM28 and interacting KRAB-ZNFs control self-renewal of human pluripotent stem cells through epigenetic repression of pro-differentiation genes. Stem Cell Reports *9*, 2065–2080.

Rowe, H.M., Jakobsson, J., Mesnard, D., Rougemont, J., Reynard, S., Aktas, T., Maillard, P.V., Layard-Liesching, H., Verp, S., Marquis, J., et al. (2010). KAP1 controls endogenous retroviruses in embryonic stem cells. Nature *463*, 237–240.

Sadate-Ngatchou, P.I., Payne, C.J., Dearth, A.T., and Braun, R.E. (2008). Cre recombinase activity specific to postnatal, premeiotic male germ cells in transgenic mice. Genesis *46*, 738–742.

Sampath Kumar, A., Seah, M.K.Y., Ling, K.Y., Wang, Y., Tan, J.H.L., Nitsch, S., Lim, S.L., Lorthongpanich, C., Wollmann, H., Low, D.H.P., et al. (2017). Loss of maternal Trim28 causes male-predominant early embryonic lethality. Genes Dev. *31*, 12–17.

Schindelin, J., Arganda-Carreras, I., Frise, E., Kaynig, V., Longair, M., Pietzsch, T., Preibisch, S., Rueden, C., Saalfeld, S., Schmid, B., et al. (2012). Fiji: an open-source platform for biological-image analysis. Nat. Methods *9*, 676–682.

Seah, M.K.Y., and Messerschmidt, D.M. (2018). From germline to soma: epigenetic dynamics in the mouse preimplantation embryo. Curr. Top. Dev. Biol. *128*, 203–235.

Uhlen, M., Fagerberg, L., Hallstrom, B.M., Lindskog, C., Oksvold, P., Mardinoglu, A., Sivertsson, A., Kampf, C., Sjostedt, E., Asplund, A., et al. (2015). Proteomics. Tissue-based map of the human proteome. Science *347*, 1260419.

Wang, D.-Z., Zhou, X.-H., Yuan, Y.-L., and Zheng, X.-M. (2010). Optimal dose of busulfan for depleting testicular germ cells of recipient mice before spermatogonial transplantation. Asian J. Androl. *12*, 263–270.

Weber, P., Cammas, F., Gerard, C., Metzger, D., Chambon, P., Losson, R., and Mark, M. (2002). Germ cell expression of the transcriptional co-repressor TIF1beta is required for the maintenance of spermatogenesis in the mouse. Development *129*, 2329–2337.

White, D.E., Negorev, D., Peng, H., Ivanov, A.V., Maul, G.G., and Rauscher, F.J., 3rd. (2006). KAP1, a novel substrate for PIKK family members, colocalizes with numerous damage response factors at DNA lesions. Cancer Res. *66*, 11594–11599.

Whitelaw, N.C., Chong, S., Morgan, D.K., Nestor, C., Bruxner, T.J., Ashe, A., Lambley, E., Meehan, R., and Whitelaw, E. (2010). Reduced levels of two modifiers of epigenetic gene silencing, Dnmt3a and Trim28, cause increased phenotypic noise. Genome Biol. *11*, R111.

Wolf, G., Yang, P., Fuchtbauer, A.C., Fuchtbauer, E.M., Silva, A.M., Park, C., Wu, W., Nielsen, A.L., Pedersen, F.S., and Macfarlan, T.S. (2015). The KRAB zinc finger protein ZFP809 is required to initiate epigenetic silencing of endogenous retroviruses. Genes Dev. *29*, 538–554.

Yoshida, S., Sukeno, M., Nakagawa, T., Ohbo, K., Nagamatsu, G., Suda, T., and Nabeshima, Y.-i. (2006). The first round of mouse spermatogenesis is a distinctive program that lacks the self-renewing spermatogonia stage. Development *133*, 1495–1505.

Zamudio, N.M., Chong, S., and O'Bryan, M.K. (2008). Epigenetic regulation in male germ cells. Reproduction *136*, 131–146.

Zhang, Z., Kostetskii, I., Moss, S.B., Jones, B.H., Ho, C., Wang, H., Kishida, T., Gerton, G.L., Radice, G.L., and Strauss, J.F., 3rd. (2004). Haploinsufficiency for the murine orthologue of Chlamydomonas PF20 disrupts spermatogenesis. Proc. Natl. Acad. Sci. U S A *101*, 12946–12951.

Stem Cell Reports, Volume *14*

Supplemental Information

Infertility-Causing Haploinsufficiency Reveals TRIM28/KAP1 Requirement in Spermatogonia

Joel H.L. Tan, Heike Wollmann, Ans M.M. van Pelt, Philipp Kaldis, and Daniel M. Messerschmidt

Tan et al. Fig. S1

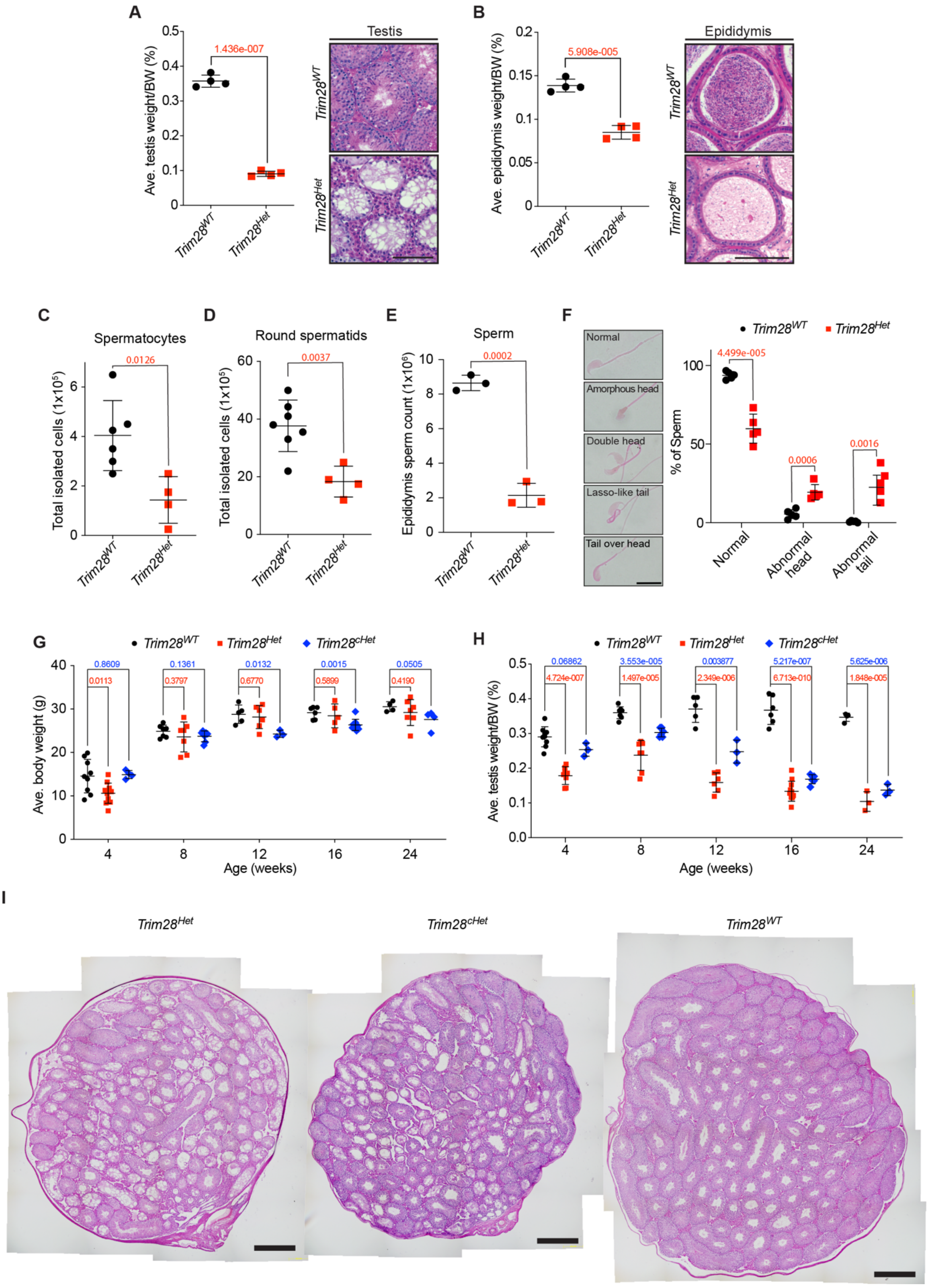

Figure S1. ***Trim28*** **haploinsufficiency phenotype (Related to Figure 1).** (A and B) H&E staining of testis (A) and epididymis (B) sections, and their respective average organ-to-body weight ratio, of males at the end of the mating experiment. Scale bar, 50 µm. SD and p values are indicated; $n = 4$. (C, D, and E) Cell counts of STAPUT-isolated spermatocytes (C) and round spermatids (D), and epididymal sperm (E) from $Trim28^{WT}$ and $Trim28^{Het}$ males at 16 weeks. SD and p values are indicated; $n \geq 3$. (F) Sperm morphological defects and their percentages observed in $Trim28^{Het}$ males at 16 weeks. Scale bar, 5 µm. SD and p values are indicated; $n = 5$. (G and H) Average body weight (G) and average testis-to-body weight ratio (H) of 4-, 8-, 12-, 16-, and 24-week-old males. SD and p values are indicated; $n \geq 3$. (I) H&E staining of whole testis sections from 25-week-old males. Scale bar, 200 µm. Student's t test was used for all significance tests.

Tan et al. Fig. S2

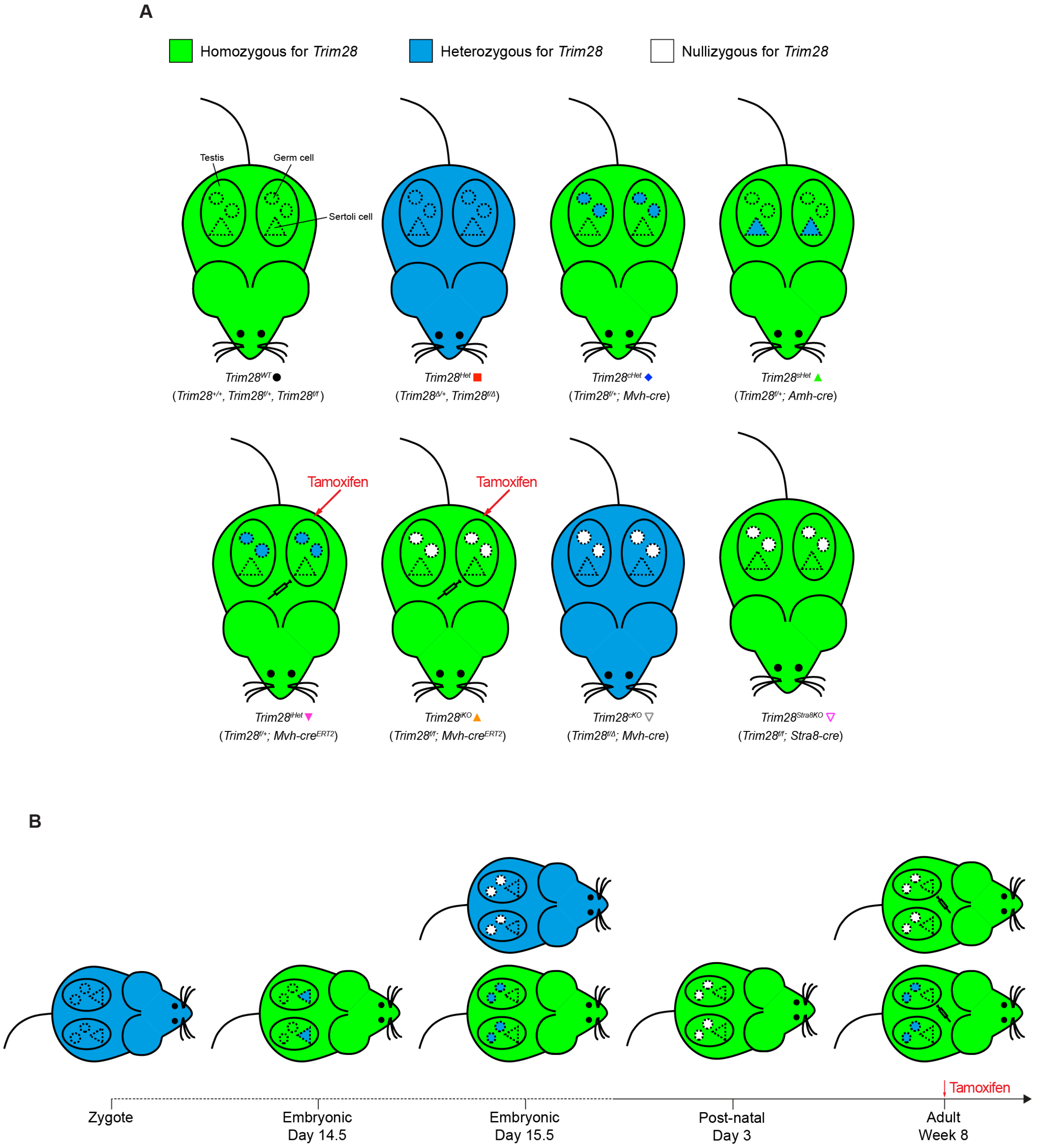

Figure S2. Mouse models. (A) Illustration of mouse models used in the study showing zygosity of *Trim28* in different cell types. Genotypes of mice as indicated. (B) Time points at which deletion of *Trim28* allele(s) occurs for the different mouse models.

Tan et al. Fig. S3

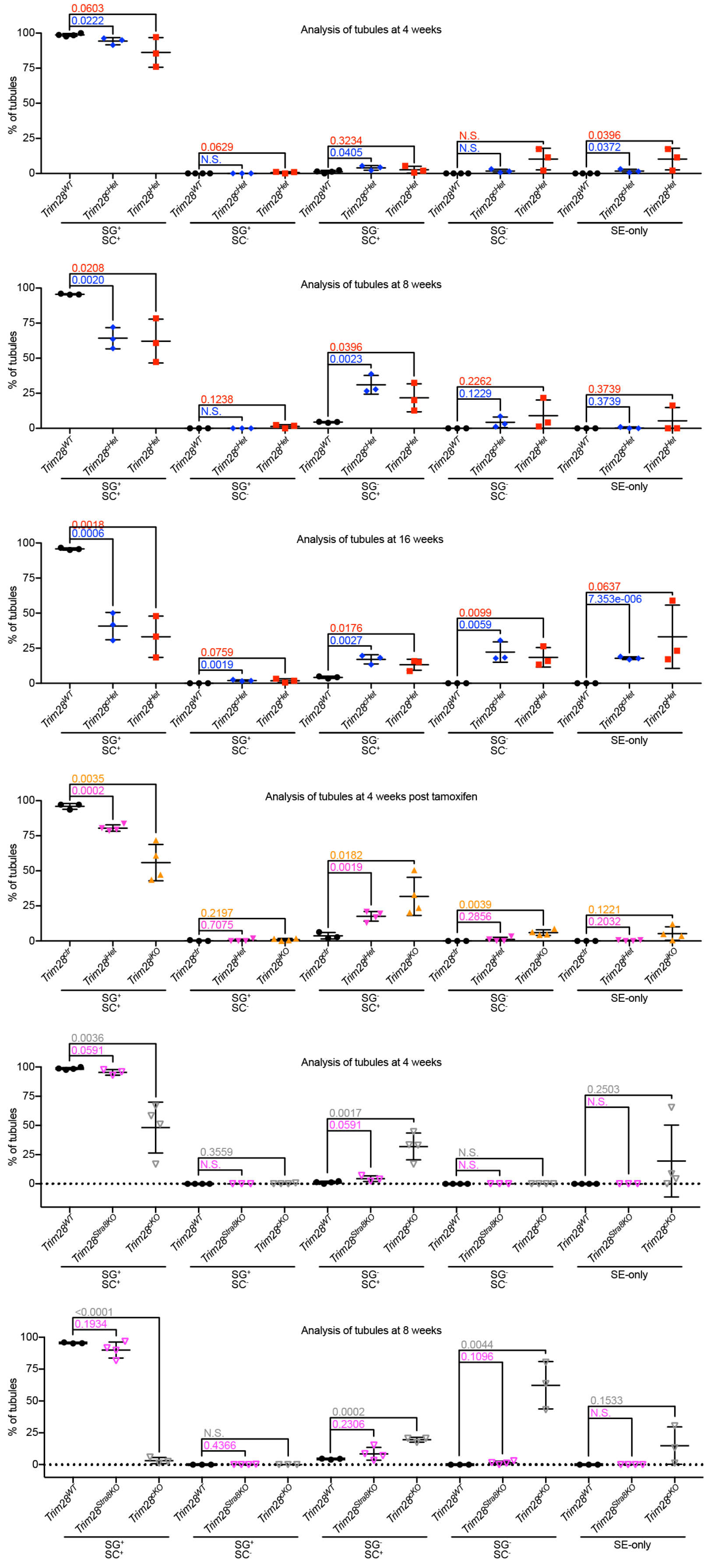

Figure S3. Statistics for sequential loss of germ cells (Related to Figures 2, 5 and 6). Germ cell composition of seminiferous tubules commonly observed in mutant and control testes. For instance, SG^{-}/SC^{+} are tubules that lack SALL4-positive spermatogonia (SG) but contain spermatocytes (SC). All tubules except Sertoli cell-only (SE-only) ones contain spermatids. SD and p values are indicated; $n \geq 3$. Student's t test was used for all significance tests.

Tan et al. Fig. S4

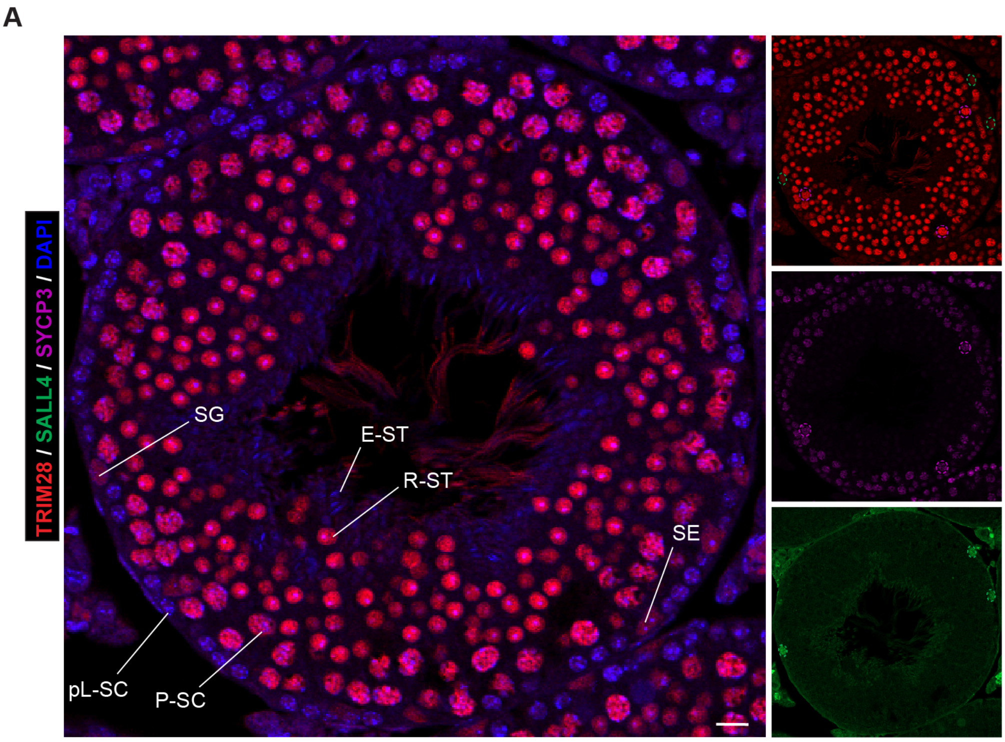

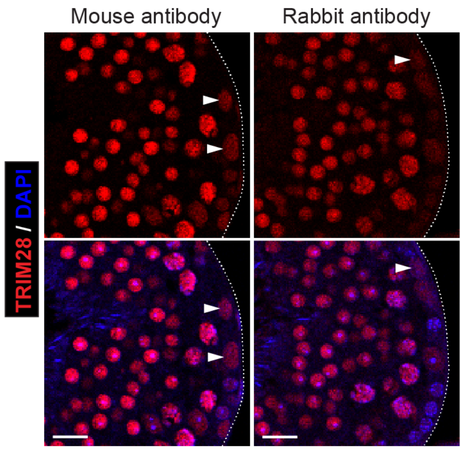

C

	Progenitor	SSC
Trim28	295.5	244.8
Ddx4	201.9	247.3
Id4	6.0	49.4
Gfrα1	5.2	85.4
Etv5	6.2	86.1
Bcl6b	6.9	64.6
Lhx1	0.5	19.4
T	0.3	13.8
Sphk1	8.1	58.7
Nanos2	1.0	4.2
Plzf	42.3	79.1
Utf1	7.9	5.7
Sall4	57.1	95.7
Stra8	133.9	60.8
c-kit	108.2	24.2
Sox9	0.4	1.3
Gapdh	419.0	422.0
Ppia	988.6	1028.0
β-actin	636.8	728.8

FPKM 500 1000

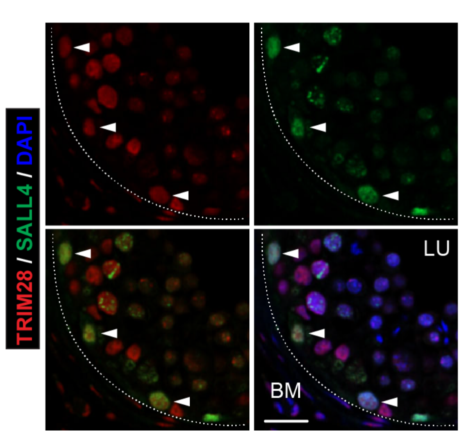

Figure S4. Expression pattern of TRIM28 (Related to Figure 3). (A) Immunofluorescence staining of a Stage 7 adult seminiferous tubule showing TRIM28 expression in germ cells up to round spermatids (R-ST). Scale bar, 20 µm. Spermatocytes (SYCP3-positive) and spermatogonia (SALL4-positive) both express TRIM28 although at varying levels (small panels). (B) Verification of TRIM28 expression in SALL4-positive spermatogonia (arrowhead) using different TRIM28 antibodies: mouse (ab22553, Abcam, left panels) and rabbit (ab10484, Abcam, right panels). Scale bars, 20 µm. (C) RNA-seq data adapted from (Helsel et al. 2017) showing expression level of *Trim28* with respect to other cellular markers in progenitor spermatogonia (ID4-eGFP dim) and SSCs (ID4-eGFP bright). (D) Immunofluorescence staining of adult human seminiferous tubule showing TRIM28 expression in SALL4-positive spermatogonia (arrowhead). Scale bar, 20 µm. BM, basement membrane; LU, lumen.

Tan et al. Fig. S5

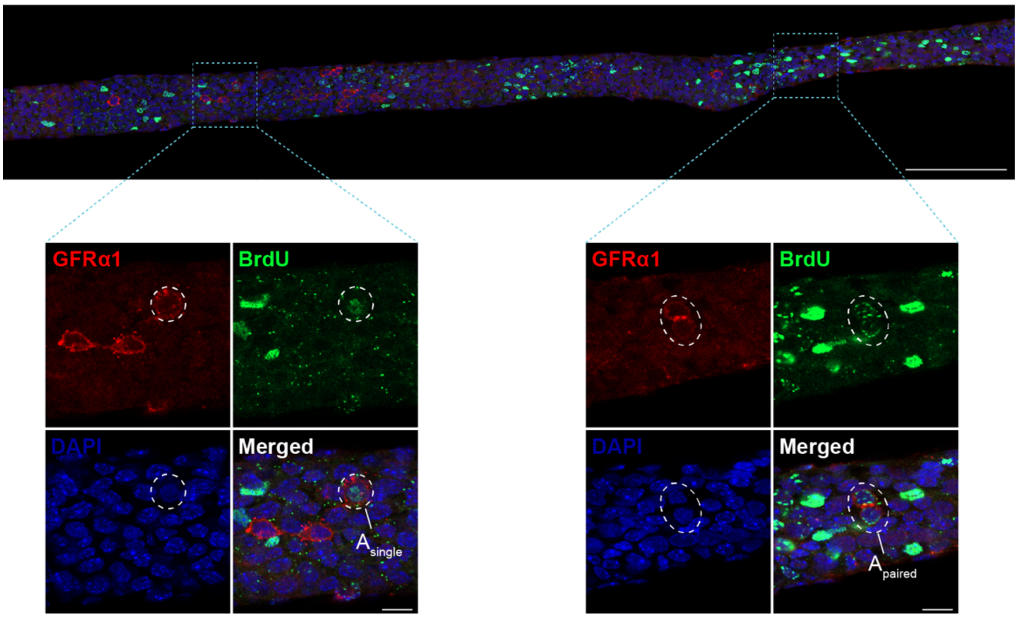

Figure S5. Whole-mount immunofluorescence staining (Related to Figure 4). Representative image of a segment of $Trim28^{WT}$ seminiferous tubule 5 days after BrdU injection. Scale bar, 100 µm. Magnified panels show BrdU-positive A_{single} and A_{paired} GFRα1-positive spermatogonia. Scale bar, 10 µm.

Tan et al. Table S1

Gene/Retrotransposon	Sequence	UPL Probe
Trim28	cgcatgtatcaggcatgaag	83
	cttccaggaaagaccttgaaga	
β-actin	aaggccaaccgtgaaaagat	56
	gtggtacgaccagaggcatac	

Table S1. Primers for RT-qPCR.

Tan et al. Table S2

**Primary antibodies**			
**Antibody**	**Company**	**Cat. No.**	**Dilution**
anti-TRIM28	Abcam	ab22553	1:200
anti-TRIM28	Abcam	ab10484	1:200
anti-SALL4	Santa Cruz	sc101147	1:100
anti-SYCP3	Santa Cruz	sc20845	1:100
anti-SOX9	Merck Millipore	ab5535	1:100
anti-BrdU	BD Biosciences	bd555627	1:100
anti-GFRα1	R&D Systems	AF560	1:100
anti-ID4	CalBioreagents	M106	1:100
**Secondary antibodies**			
**Antibody**	**Company**	**Cat. No.**	**Dilution**
Donkey anti-Mouse Alexa Fluor 488	Thermo Fisher	A32766	1:500
Donkey anti-Rabbit Alexa Fluor 594	Thermo Fisher	A32754	1:500
Donkey anti-Goat Alexa Fluor 647	Thermo Fisher	A32849	1:500

Table S2. Antibodies for immunofluorescence staining.

Supplemental Experimental Procedures

Epididymal spermatozoon count

Incisions were made in both caudal epididymides (left and right) before placing them in DSP buffer (0.9% NaCl and 0.05% Triton™ X-100) for 15 min to allow spermatozoa to swim out. A 1:10 dilution of spermatozoa suspension was made before loading both chambers of the hemocytometer (Neubauer Chamber). Spermatozoa were quantified using five squares of each chamber. The counts from both chambers were averaged and the final spermatozoon count was determined using the formula: Spermatozoon count = Dilution × (Count in 5 squares) × 0.05 × 10^6.

H&E spermatozoa smear

Caudal epididymides were snipped and placed in 1 mL PBS at 37°C for 30 min. Spermatozoa released were centrifuged at 600 × *g* for 10 min and resuspended in a smaller volume of PBS before smearing onto glass slides. Slides were air-dried overnight at room temperature. Prior to H&E staining (haematoxylin step onwards), slides were rehydrated in water. Spermatozoon morphology was analysed by counting at least ten 40X magnification fields of spermatozoa. A minimum of three mice were examined for each genotype.
